# Presence of Legacy and Emerging PFAS in Human Liver Specimens Banked in the United States from 2000 to 2024

**DOI:** 10.1021/acs.est.5c14780

**Published:** 2026-04-07

**Authors:** Juliana Agudelo Areiza, Jitka Becanova, Simon Vojta, Johanna Ganglbauer, Udayan Apte, Luigi Brunetti, Sean Kumer, Faiz Haque, Euna Kim, Elsie M. Sunderland, Rainer Lohmann, Fabian C. Fischer, Angela Slitt

**Affiliations:** † Department of Biomedical and Pharmaceutical Sciences, College of Pharmacy, 15524 University of Rhode Island, Kingston, Rhode Island 02881, United States; ‡ Graduate School of Oceanography, 54083 University of Rhode Island, Narragansett, Rhode Island 02881, United States; § Department of Pharmacology, Toxicology and Therapeutics, 4202 University of Kansas Medical Center, Kansas City, Kansas 66103, United States; ∥ Pharmacy Practice and Administration, Ernest Mario School of Pharmacy, 5970 Rutgers University, Piscataway, New Jersey 08854, United States; ⊥ Department of Surgery, 302759 University of Kansas Medical Center, Kansas City, Kansas 66103, United States; # Harvard School of Engineering and Applied Sciences, 1812 Harvard University, Boston, Massachusetts 02115, United States; ∇ University of Vienna, Wien 1010, Austria

**Keywords:** PFAS, hepatic accumulation, temporal trends, biomonitoring, demographics

## Abstract

Per- and polyfluoroalkyl substances (PFAS) are persistent, bioaccumulative chemicals linked to liver toxicity and metabolic disease. 54 PFAS were measured in 211 adult human livers collected between 2000 and 2024 to reveal temporal trends, relative PFAS abundance, and demographic predictors of hepatic burden. PFAS were detected in 210 individuals, with 15 compounds found in ≥30 livers. Total summed PFAS concentrations decreased by 94% over the 24-year period in weighted linear regression, and by 68% after adjusting for age, sex, and liver health in multivariate models. Since 2019, a ∼950-fold variability in concentration was observed, and the PFAS profile in the liver shifted from sulfonates and carboxylates to proportionally more sulfonamides and fluorotelomers. Sampling year was the strongest predictor of hepatic PFAS concentration in multivariate models. Age was positively associated with several long-chain PFAS, which is consistent with years-long elimination half-lives. Males had higher perfluoroundecanoic acid, perfluorododecanoic acid, and 9-chlorohexadecafluoro-3-oxanonane-1-sulfonic acid concentrations, whereas females had higher 8:2 fluorotelomer sulfonic acid concentrations. Nonalcoholic fatty liver disease was associated with lower concentrations of seven PFAS. While legacy PFAS declined following phaseouts, other PFAS increasingly drive liver burdens, with our data showing targeted PFAS comprise <10% of extractable organofluorine, highlighting the inadequacy of substance-by-substance regulatory approaches.

## Graphical abstract

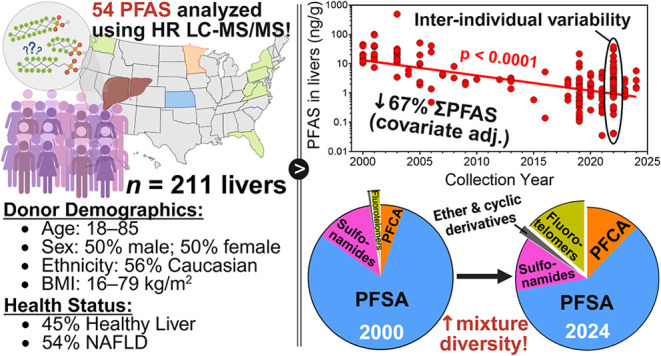

## 1 Introduction

Per- and polyfluoroalkyl substances (PFAS) are a diverse class of synthetic chemicals widely used for their resistance to heat, water, and degradation.^1,2^ Their widespread use has led to near-universal human exposure, with detectable serum levels in 98–99% of U.S. individuals.^3,4^ Legacy PFAS, such as perfluorooctanoic acid (PFOA) and perfluorooctanesulfonic acid (PFOS), have been linked to cancer,^5^ immunotoxicity,^6^ thyroid dysfunction,^7^ and developmental effects.^8,9^ The liver is a key target organ for PFAS accumulation and toxicity, with evidence linking PFAS exposure to hepatocellular injury,^10^ steatosis,^11^ and dysregulated lipid metabolism in humans.^12,13^ The phase out of legacy PFAS has driven substitution with structurally diverse replacement PFAS,^14^ most of which lack human exposure data. While serum PFAS levels are well characterized through monitoring efforts,^15–18^ data on PFAS levels in organs such as the liver remain sparse, a gap this study aimed to address.

Legacy PFAS, such as PFOA and PFOS, are highly persistent in humans, with serum elimination half-lives of 1.3 to 8.5 years.^19–21^ These compounds preferentially accumulate in liver tissue, with PFOS concentrations in human liver tissue averaging 6.3 ng/g—approximately 4-fold higher than in blood and 2–3 times higher than in kidneys, lungs, and spleen.^22^ This enrichment likely reflects facilitated uptake by solute carrier transporters^23–25^ and retention driven by the strong affinity of long-chain PFAS for phospholipids^26,27^ and the high phospholipid content of the liver (∼2.5% v/v).^28^

Some PFAS with perfluorinated carbon chain lengths of 7 or more (η_*pfc*_ ≥ 7), such as PFOS and PFOA, have been shown to induce hepatocellular damage, elevate liver enzymes, and cause steatosis in mice,^29^ rats,^30^ and monkeys.^31^ Epidemiological studies support these findings, linking η_*pfc*_ ≥ 7 PFAS exposure to elevated serum liver enzymes,^11^ elevated serum total cholesterol and LDL-cholesterol,^32^ and hepatic fat accumulation in humans.^33^ A recent meta-analysis of 24 human studies found associations between PFOS exposure and higher alanine aminotransferase (ALT) values.^34^ Notably, 9-chlorohexadecafluoro-3-oxanonane-1-sulfonic acid (9Cl-PF3ONS, η_*pfc*_ = 8), an emerging PFOS substitute recently found in the serum of Chinese populations,^35^ has been linked to hepatotoxicity in rodents,^36^ underscoring the need to monitor emerging PFAS accumulation in human livers. Despite clear evidence that the liver is a primary target of PFAS toxicity, only four studies have quantified up to 21 predominantly legacy PFAS in human livers,^22,37–39^ all with limited sociodemographic and geographic coverage.

Human biomonitoring efforts typically focus on serum or plasma^15–18^ and a limitation of only surveying exposure through blood is that it might not fully capture exposure to tissues. Growing evidence indicates that specific PFAS are transported into the liver via Organic Anion Transporting Polypeptide (OATP)-mediated uptake.^23,24,40^ In matched serum:liver samples from adolescents with liver disease, perfluoroheptanoic acid (PFHpA, η_*pfc*_ = 6) showed high hepatic accumulation relative to longer-chain PFAS (η_*pfc*_ ≥ 8).^39^ These findings indicate that serum-based measurements may underestimate hepatotoxicity risk, particularly for PFAS that are preferentially transported into and retained in hepatic tissue, with potential variation in uptake and retention mechanisms across age-related renal function,^41^ reproductive elimination,^42^ and hepatic tissue composition in fatty liver disease.^43^

The overarching aim of this study was to quantify PFAS concentrations in livers from individuals in the United States and assess whether hepatic PFAS profiles have shifted from 2000 to 2024 due to legacy phaseouts and the emergence of replacement compounds. A highly sensitive solid-phase extraction liquid chromatography high-resolution mass spectrometry (SPE-LC-HRMS) method using ENVI-Carb sorbent was developed to quantify a total of 54 PFAS, including legacy compounds, emerging alternatives (e.g., 9Cl-PF3ONS), and precursors. Banked liver samples (*n* = 211) collected over two decades were obtained from adults aged 18–85 years. The sampling region included areas with major PFAS manufacturing and extensive contamination. Nationally, more than 9,300 PFAS-contaminated sites have been reported,^44^ with drinking water contamination and military sources common in the states represented here, and particularly high counts in NY, MN, PA, and FL. Regression analyses were utilized to assess temporal trends in hepatic PFAS and evaluate the influence of sampling year, age, sex, and liver health status on individual PFAS and total liver burdens. Extractable organofluorine (EOF) was quantified in a subset of liver samples to determine the fraction of organofluorine not explained by targeted PFAS. Results were used to better understand exposure sources and potential links to liver disease.

## 2 Materials and Methods

### 2.1 Chemicals and Solvents

Liquid chromatography–mass spectrometry (LC-MS)-grade methanol was obtained from Honeywell. LC-MS-grade water, acetonitrile, and ammonium hydroxide were obtained from Fisher Chemical. Formic acid and sodium fluoride (NaF) were obtained from ACROS Organics. Analytical standards for LC-HRMS analysis (Table S1 and S2, Supporting Information) were obtained from Wellington Laboratories.

### 2.2 Specimen Demographics

A total of 211 banked, deidentified human liver specimens were acquired under Institutional Review Board (IRB)-approved protocols from several tissue banks located in the United States (Figure S1). IRB exemption for the use of biospecimens from participants that cannot be reidentified was granted by the University of Rhode Island Institutional Review Board to Dr. Angela Slitt (IRB1112–132, IRB2324–106). The liver specimens obtained from the University of Kansas Medical Center (KUMC) were taken during liver resection surgeries under an approved IRB protocol and shared with Dr. Slitt via a material transfer agreement. The study protocol was reviewed and approved by the Institutional Review Board of KUMC (approval #11378). All participants gave their informed consent. An additional 88 liver samples obtained from tissue donors were provided by the University of Minnesota Liver Tissue Cell Distribution System (UMN LTCDS). The remaining 35 samples were purchased as deidentified tissue specimens from the National Disease Research Interchange (NDRI) by Dr. Luigi Brunetti (Rutgers Biomedical Health Sciences Institutional Review Board, Pro2019001020) and shared with Dr. Slitt under a material transfer agreement and IRB approval. Deidentified details of donor demographics for the specimens collected from KUMC (Lawrence, KS, U.S.), UMN LTCDS (Minneapolis, MN, U.S.; RRID:SCR_004840), and NDRI (Philadelphia, PA, U.S.; RRID:SCR_000550) are provided in Table 1.

**Table 1:** Donor Specimen Demographics (*n* = 211)

**Age mean ± SD (Range,** *n* **)**	57.51 ± 13.53 (18–85, 210)
**Sex,** *n* **(%)**	
Male	105 (49.76%)
Female	106 (50.24%)
**Ethnicity,** *n* **(%)**	
Caucasian	119 (56.4%)
Black or African American	8 (3.8%)
American Indian or Alaska Native	4 (1.9%)
Asian	2 (<1%)
Other/Unknown	78 (36.96%)
**Collection Year Range,** *n* **(%)**	2000–2010 (81, 38.39%)
	2012–2024 (127, 60.19%)
	Unknown (3, 1.42%)
**BMI** ^a^ **(kg/m** ^**2**^ **), mean ± SD (Range,** *n* **)**	31.27 ± 9.17 (16.64–79.38, 118)
**Health Status,** *n* **(%)**	
Normal	95 (45%)
NAFLD^b^	114 (54%)
Unknown Diagnosis	2 (<1%)
**Tissue Bank Specimen Location,** **donor,** *n* **(%)**	
LTCDS^c^	MN, 88 (41.71%)
KUMC^d^	PA (14, 6.64%), FL (7, 3.32%), VA (6, 2.84%), NY (5, 2.37%), WV (2, <1%), and WA (1, <1%)
NDRI^e^	KA, 88 (41.71%)

a BMI = Body mass index.

b NAFLD = Nonalcoholic fatty liver disease.

c LTCDS = Liver Tissue Cell Distribution System, University of Minnesota.

d KUMC = University of Kansas Medical Center.

e NDRI = National Disease Research Interchange.

The donor cohort included 105 males and 106 females, with a mean age of 57.5 years (range: 18–85; Table 1). The self-reported ethnicity distribution was 56% Caucasian, 3.8% Black or African American, 1.9% American Indian or Alaska Native, <1% Asian, and 37% unidentified. The mean body mass index (BMI) was 31 kg/m^2^ (range: 17–79), based on available data for 118 of the 211 liver specimens. Clinical classifications indicated that 45% of livers had no diagnosed pathology, 54% were categorized as nonalcoholic fatty liver disease (NAFLD), and <1% had an unknown diagnosis. Collection years were available for 208 specimens: 81 were collected between 2000 and 2010, 127 between 2012 and 2024, and 3 had unreported dates.

### 2.3 Sample PFAS Extraction

Prior to sample preparation, all equipment was rinsed two to three times with 3% ammonium hydroxide in methanol, followed by methanol, to minimize background PFAS contamination from consumables. Human liver specimens (*n = 211*), matrix-matched positive verification controls (rat liver, *n = 24)*, and procedural negative controls (100% LC/MS-grade methanol, *n =* 42) were prepared according to a modified EPA Method 1633. This method is performance-based and permits modifications, provided that all quality assurance/quality control (QA/QC) criteria are satisfied. Approximately 0.5 g of preweighed frozen human liver tissue, ∼0.5 g of positive verification controls, and 0.5 mL of procedural negative controls were spiked with 4 ng of an extractable internal standard (EIS). Additionally, positive verification controls were also spiked with 4 ng of the native standard mixture. Internal and native standards are detailed in Table 1 of the Supporting Information. Equipment cleanup, reference matrix use, isotopically labeled standard spikes [EIS and nonextracted internal standard (NIS)], carbon cartridge cleanup, and overall QA/QC followed EPA Method 1633.

All samples were homogenized in 4 mL of LC/MS-grade 1% formic acid in methanol using 2.8 mm ceramic bead-filled 15 mL tubes on an OMNI Bead Ruptor Elite (Omni International, Kennesaw, GA), applying the manufacturer’s preset program for liver tissue (15 mL tube setting, 30 s at 6.00 m/s). Homogenates were sonicated for 1 h to enhance extraction efficiency, followed by centrifugation at 4000 rpm for 10 min at 4 °C. The resulting supernatant was carefully transferred into a precleaned 15 mL polypropylene tube and set aside for subsequent processing. The remaining protein pellet in the OMNI tube was extracted with 4 mL of ice-cold LC/MS-grade 1% ammonium hydroxide in methanol, homogenized on the OMNI Bead Ruptor Elite (one cycle), sonicated for 30 min, and centrifuged. The second supernatant was transferred to a 15 mL polypropylene tube containing the initial extract in 1% formic acid in methanol. A third extraction was performed on the residual pellet using 4 mL of ice-cold LC/MS-grade methanol, following the same homogenization, sonication, and centrifugation procedure. The final pooled extract (∼11 mL) consisted of a 1% formic acid in methanol (v/v) supernatant, 1% ammonium hydroxide in methanol (v/v) supernatant, and 100% methanol supernatant. Tissue extractions under acidic, basic, and neutral solvent conditions were optimized to extract a broader range of PFAS. The combined extract was vortexed for 30 s, centrifuged to remove residual protein, and transferred to a precleaned 15 mL polypropylene tube. The solvent was then concentrated under a gentle nitrogen stream at 36 °C to ∼1.5 mL in preparation for graphitized carbon solid-phase extraction (SPE) using Superclean ENVI-Carb SPE cartridges (Millipore Sigma) for sample cleanup, as detailed in Section 4 of the Supporting Information. Carbon cleanup provided sufficient matrix removal while minimizing the low surrogate and analyte recoveries associated with WAX SPE.

Next, samples were evaporated to ∼0.5 mL and transferred to a precleaned 1.7 mL Eppendorf tube. The original polypropylene tube was rinsed with ∼500 μL of LC/MS-grade 100% methanol to ensure quantitative transfer, and the rinsing was combined with the sample. The combined sample was evaporated again to ∼0.5 mL, spiked with 4 ng of NIS, and vortexed for 30 s. Samples were then centrifuged at 10,000 rpm for 30 s, and a 40 μL aliquot was reconstituted with 10 mM ammonium acetate in water to yield a final solvent composition of 40:60 (methanol:water).

### 2.4 Instrumental Analysis

Instrumental analysis was conducted on a SCIEX ExionLC AC UHPLC system coupled to a SCIEX X500R quadrupole time-of-flight tandem mass spectrometer (QTOF-MS/MS) at the University of Rhode Island. Analyte separation was achieved using a Phenomenex Gemini C18 analytical column (3 μm, 110 Å, 50 × 2 mm) preceded by a Phenomenex SecurityGuard cartridge. To mitigate the PFAS background from the instrument, a second Phenomenex Gemini C18 column (5 μm, 110 Å, 50 × 4.6 mm) was installed in-line to serve as a delay column. The aqueous mobile phase (MP A) consisted of 10 mM ammonium acetate in water, and the organic mobile phase (MP B) consisted of 10 mM ammonium acetate in methanol. LC operating parameters were as follows: flow rate, 0.3 mL/min; injection volume, 20 μL; and column oven temperature, 45 °C. The solvent gradient for MP B increased from 40% to 80% over 1–5.5 min, then from 80% to 100% over 5.5–7 min, was held at 100% for 1 min, returned to 40% from 8 to 8.5 min, and was held at 40% for an additional 6.5 min.

Quantification of target analytes was performed using a high-resolution tandem mass spectrometry (HR MS/MS) method with high-resolution multiple reaction monitoring (HR MRM). Negative electrospray ionization (ESI) was applied with the following parameters: curtain gas at 30 psi, ion source gas one at 40 psi, ion source gas two at 60 psi, and source temperature at 450 °C. Detailed MS/MS parameters for each targeted compound are provided in Section S2 of the Supporting Information. For most compounds, the MS/MS channel detecting fragmented ions was used for quantification; for selected compounds (PFOA, branched perfluorohexanesulfonic acid [Br-PFHxS], linear PFHxS [L-PFHxS], branched PFOS [Br-PFOS], and linear PFOS [L-PFOS]), the HRMS channel was used to account for matrix effects. Each sample was run once on the instrument and analyzed twice—once for a core set of 29 target analytes and once for an additional set of 25 extended target analytes, as indicated in Table S2 of the Supporting Information.

### 2.5 Quality Assurance/Quality Control (QA/QC)

Quantification of all targeted PFAS in samples and quality control (QC) samples was performed using an isotope dilution method with a matrix-matched calibration curve. Concentrations were recovery-corrected using mass-labeled extractable internal standards (Tables S2–S5 of the Supporting Information) spiked into each sample prior to extraction. Instrumental QC was maintained by analyzing a continuing calibration verification (CCV; mid-calibration standard) with every batch of samples. Recoveries of all targeted compounds in the positive controls were within the modified QC acceptance limits (Table S4 of the Supporting Information). A clean solvent was run after each positive control sample to monitor carryover.

To ensure laboratory quality control, a total of 42 procedural negative controls were included throughout the extraction procedure to monitor background PFAS levels. All reported sample concentrations were blank-corrected by using the average of a subset of procedural negative controls. Blank corrections were applied only when the original value was greater than the method detection limit (MDL). Method detection limits were determined as follows: when no analyte signal was detected in the method blanks, the instrumental detection limit (IDL) was used, defined as the concentration producing a signal-to-noise ratio of 10 in the sample matrix. When analytes were detected in the procedural negative controls, the MDL was calculated as the mean blank concentration plus three times the standard deviation. MDLs for all targeted PFAS are provided in Table S6 of the Supporting Information.

Herein, a modified EPA Method 1633A was implemented for QC/QA, with a threshold for EIS recovery criteria specified in Table S3 of the Supporting Information. For all samples, the retention time difference between analytical standards and their corresponding EIS did not exceed 0.1 or 0.4 min when the EIS had a different molecular structure from the analyte. In the latter case, the retention time difference was corrected using the average shift between the analytical standard and its corresponding EIS in the CCV. For all target analytes, the ion abundance ratio was within −100% to 100%. The ion abundance ratio was calculated as the ratio of the peak area in the HRMS (confirmation) channel to that in the HRMS/MS (quantification) channel, normalized to the average of the same ratio in the CCV quality control samples, and offset by one, such that zero indicated no deviation between the two channels. QA/QC evaluation for retention time shifts, method detection limits, recovery rates, and ion abundance ratios was performed in Jupyter notebooks.^45^ Samples and compounds failing these criteria were flagged and excluded from further analysis. Of the 211 livers measured, only one liver (<1%) was excluded from further analysis, as it resulted in 42 compounds (78%) not being detected and 12 (22%) measuring below the detection limit—including PFOA and PFOS.

### 2.6 Extractable Organofluorine Analysis

Preliminary data on EOF concentrations were derived for a subset of liver specimens (*n* = 8) from the early 2000s, selected to match donor age, sex, and geographic origin, including two donor-matched samples. EOF analyses used acetonitrile tissue extractions with 1 g of wet-weight subsamples, following previously published methods.^46^ Bovine liver was used as a control and spiked with inorganic fluorine as sodium fluoride (10 mg F/L; negative control) and a PFAS mixture (*n* = 4, 9,948 ng F/mL; positive control). Controls were equilibrated for at least 1 h prior to homogenization. All samples and spiked controls, DI water procedural blanks (*n* = 2), and matrix extraction blanks (*n* = 2) were homogenized in 5 mL acetonitrile using 4.8 mm stainless steel bead-filled 15 mL polypropylene tubes on an MP Biomedicals FastPrep-24 Homogenizer, followed by centrifugation at 4000 rpm for 5 min at room temperature (repeated twice). Combined supernatants (∼10 mL) were frozen overnight at −20 °C to allow for lipid precipitation and then transferred to new polypropylene tubes after centrifugation (4000 rpm for 3 min at room temperature). Extracts were concentrated under a gentle nitrogen stream to near dryness, reconstituted in 0.5 mL of LC/MS-grade methanol, and subjected to ENVI-Carb cleanup (∼25 mg of dispersive ENVI-Carb and 25 μL of acetic acid).

A 450 μL aliquot of the extracts was transferred to combustion ion chromatography (CIC) vials for EOF analysis using a Metrohm CIC system equipped with a combustion unit from Analytik Jena, a 920 Absorber Module, and a 930 Compact IC Flex ion chromatograph (Metrohm). Fluoride (F^–^) was quantified by ion conductivity. Sample concentrations exceeding the instrumental background were corrected by subtracting the mean of DI water procedural blanks per batch. MDLs were defined as three times the standard deviation of matrix extraction blanks, adjusted by the dilution factor.

Recoveries of all targeted compounds in the positive controls were within acceptable percentages (Table S7 of the Supporting Information). EOF batch method detection limits are summarized in Table S8 of the Supporting Information. To estimate the fraction of EOF explained by targeted PFAS, LC-HRMS-derived PFAS concentrations (*n* = 11) were converted to fluorine equivalents for direct comparison. EOF and targeted PFAS concentrations were matched by donor demographics, as summarized in Table S9 of the Supporting Information.

### 2.7 Statistics

Weighted linear regression of the log-transformed sum of PFAS concentrations against the year of specimen collection was performed to evaluate temporal trends in PFAS liver concentrations. To account for differences in the number of samples collected per year, each data point was weighted by the inverse of that year’s sample size. This approach prevents years with more samples from dominating the regression and reduces bias due to unequal sampling across the study period. Livers with ambiguous sampling dates were excluded, resulting in a final data set of *n* = 178 livers with clearly defined collection years for this analysis. The final model takes the form log_10_(PFAS) = β_0_ + β_1_ × Year. To compare across chemical classes, each year’s concentrations were normalized to the total ∑PFAS, and individual compounds were expressed as percent contributions. These temporal trends were analyzed both for individual targeted analytes and aggregated by the PFAS subclass to visualize shifts in composition over time.

To identify predictors of PFAS presence in liver tissue, a multivariate linear regression (MLR) model using log_10_-transformed total PFAS and individual PFAS concentrations detected in ≥30 livers (*n* = 15 compounds plus ∑PFAS, including branched and linear isomers) as the dependent variables was conducted. A detection threshold of ≥30 was chosen to ensure reliable parametric estimates while retaining analytes with heterogeneous detection patterns across the data set. This substitution method assumes values below detection follow a uniform distribution between zero and the MDL, where the expected value equals MDL/2, and is recommended by EPA guidance for environmental contaminant data.^47^ While this may introduce uncertainty for compounds with more nondetects, it ensures consistency across all PFAS. The model used a subset of 205 samples with complete metadata for all covariates: sampling year, sex, liver health status, and donor age. For samples with date ranges rather than specific years (e.g., 2000–2005), the midpoint was used (e.g., 2002.5). The independent variables included year of sample collection (continuous), age (continuous), sex (binary), and health status (binary; NAFLD vs healthy). All variables were entered simultaneously into the model.

MLR model fit was evaluated using *R* ^2^ and ANOVA-based F-statistics, with *p* < 0.05 considered statistically significant. Residual normality was assessed using Shapiro–Wilk, D’Agostino-Pearson, and Kolmogorov–Smirnov tests. Multicollinearity was assessed using variance inflation factors (VIF), which were all below the commonly used threshold of 5, indicating no critical collinearity. The highest values were observed for liver health (VIF = 1.78) and sampling year (VIF = 1.77), reflecting moderate correlation between these variables (*R* ^2^ = 0.43). This relationship was consistent with a greater proportion of NAFLD samples being collected in recent years. Other pairwise correlations were low: year and sex (*R* ^2^ = 0.03), and year and age (*R* ^2^ = 0.02), suggesting that these predictors contributed independent information to the regression model.

To enhance the interpretability of regression coefficients, β estimates were transformed into relative percent changes in liver PFAS concentrations using

1 $$\mathrm{percent change =}\left(10^{\beta }-1\right)\times 100$$

For continuous predictors (sampling year and age), β values were scaled to reflect the change over 10 years. For binary predictors (sex and liver health), percent changes represent the relative difference between the two categories (female vs male and NAFLD vs healthy).

## 3 Results and Discussion

### 3.1 Detection Frequencies and Concentration Ranges in Human Livers

In this study, 211 adult human livers collected between 2000 and 2024 were analyzed for 54 PFAS. Remarkably, PFAS were detected in all but one liver out of the 211 livers sampled (99.5% of samples). We detected 25 compounds, including linear and branched isomers, in at least three individuals (Figure 1). Legacy PFAS were frequently detected, including PFOA (detection frequency: 62%), perfluorononanoic acid (PFNA) (57%), and linear PFOS (L-PFOS) and branched PFOS (Br-PFOS) (99% and 95%, respectively). These compounds also exhibited the highest concentrations, with median levels of 0.22 ng/g for PFOA (IQR: 0.15–0.32 ng/g), 0.18 ng/g for PFNA (IQR: 0.12–0.31 ng/g), 0.15 ng/g for perfluorodecanoic acid (PFDA) (IQR: 0.12–0.26 ng/g), 1.39 ng/g for L-PFOS (IQR: 0.48–6.86 ng/g), and 0.89 ng/g for Br-PFOS (IQR: 0.28–2.93 ng/g). Notably, long-chain perfluoroalkyl carboxylic acids (PFCAs) were detected, including perfluoroundecanoic acid (PFUdA) (η_*pfc*_ = 11, detection frequency: 44%, median: 0.14 ng/g), perfluorododecanoic acid (PFDoA) (η_*pfc*_ = 12, 37%, median: 0.03 ng/g), perfluorotridecanoic acid (PFTrDA) (η_*pfc*_ = 13, 13%, median: 0.10 ng/g), and perfluorotetradecanoic acid (PFTeDA) (η_*pfc*_ = 14, 1%, median: 0.09 ng/g).

**Figure 1:**
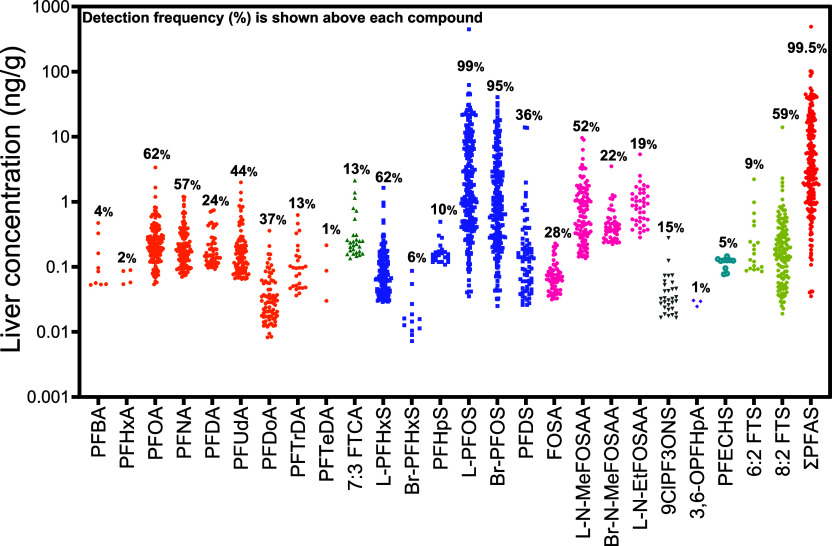
PFAS concentrations in human liver samples (*n* = 211) were collected from 2000 to 2024. Only compounds detected in ≥3 samples are shown. Numbers above each data point indicate the detection frequency (%). PFAS are color- and symbol-coded by chemical class: perfluoroalkyl carboxylic acids (PFCA, orange circles), fluorotelomer carboxylic acids (FTCA, green triangles), perfluoroalkyl sulfonic acids (PFSA, blue squares), perfluorooctanesulfonamides and derivatives (FOSAA, pink circles), chlorinated ethers (gray downward triangles), perfluoroalkyl ether carboxylic acids (PFECA, purple diamonds), cyclic perfluoroalkyl sulfonic acids (cyclic PFSA, blue open circles), fluorotelomer sulfonates (FTS, yellow circles), and total PFAS (red circles).

The short-chain perfluorobutanoic acid (PFBA, η_*pfc*_ = 3) was detected in 8 livers (4%) collected in the years 2000, 2006, 2007, 2013, and 2022—with 3 livers being collected in 2022. Despite the low molecular weight (214 g/mol) and hydrophobicity of PFBA (log *K*_ow_ = 2.23),^48^ liver concentrations were comparable to those of some long-chain PFCA. For instance, the median concentration of PFBA (0.08 ng/g) was similar to that of PFTeDA (η_*pfc*_ = 14; 0.09  ng/g) and exceeded that of PFDoA (η_*pfc*_ = 12; 0.03  ng/g). PFBA is a known degradation product of side-chain fluorinated polymers^49^ and is also used as a substitute for longer-chain perfluorocarboxylic acids in certain consumer products.^50^ PFBA has been widely detected in rainwater, groundwater, drinking water, and even Arctic ice cores, and is still actively used as a PFOA replacement.^51,52^

Among the PFOS precursors detected, linear-*N*-methyl perfluorooctanesulfonamidoacetic acid (L-N-MeFOSAA) and branched-*N*-MeFOSAA (Br-N-MeFOSAA) were found in 52% and 22% of the liver samples, with median concentrations of 0.60 ng/g (IQR: 0.32–1.4 ng/g) and 0.39 ng/g (IQR: 0.28–0.52 ng/g), respectively. Linear-*N*-Ethyl perfluorooctanesulfonamidoacetic acid (L-*N*-EtFOSAA) appeared in 19% of livers at a median of 0.87 ng/g (IQR: 0.53–1.3 ng/g). Perfluorooctanesulfonamide (FOSA) was present in 28% of samples, though at lower levels (0.07 ng/g; IQR: 0.04–0.09 ng/g). These sulfonamides were widely used in commercial applications, including textile and paper coatings, surface protectants, and insecticides.^53^ Though phased out in North America and Europe in the early 2000s, these precursors continue to leach from contaminated sites, with microbial transformation to terminal PFAS expected to persist for decades after initial use.^54^ Despite production phase outs, FOSA and L-N-MeFOSAA concentrations showed no clear decline over the study period. FOSA ranged from 0.04 to 0.13 ng/g in 2000 to 0.04–0.23 ng/g in 2022, while L-N-MeFOSAA ranged from 1.0 to 3.71 ng/g in 2000 to 0.14–9.60 ng/g in 2020.

9Cl-PF3ONS was detected in 15% of liver samples, with a median concentration of 0.03 ng/g (IQR: 0.02–0.05 ng/g), and 11-chloroeicosafluoro-3-oxaundecane-1-sulfonic acid (11Cl-PF3OUdS) was only detected in one individual at a concentration of 0.02 ng/g. These PFAS are components of F-53B, a chlorinated polyfluoroalkyl ether sulfonic acid mixture used as a mist suppressant in electroplating and as a replacement for PFOS in AFFF, particularly in China.^35^ Their detection in human liver tissue is consistent with previous findings of chlorinated ether PFAS in human breast milk in China.^55^ To the best of our knowledge, this is the first time these compounds have been detected in U.S. individuals.

Perfluoroalkyl ether carboxylic acids and cyclic perfluoroalkyl sulfonic acids were detected in a limited subset of livers. Nonafluoro-3,6-dioxaheptanoic acid (3,6-OPFHpA) was detected in 3 liver samples, with a median concentration of 0.03 ng/g (IQR: 0.027–0.030 ng/g). Perfluoroethylcyclohexanesulfonic acid (PFECHS) was detected in 5% of the samples. Despite being detected less frequently, the median concentration of PFECHS (0.13 ng/g) was comparable to those of PFHpS (median: 0.15 ng/g) and perfluorodecanesulfonic acid (PFDS, median: 0.14).

Of the fluorotelomer PFAS included in the method, 8:2 fluorotelomer sulfonic acid (8:2 FTS) was the most frequently detected, present in 59% of samples with a median concentration of 0.17 ng/g (IQR: 0.07–0.36 ng/g), followed by 6:2 fluorotelomer sulfonic acid (6:2 FTS) in 19 livers (median: 0.16 ng/g; IQR: 0.10–0.34 ng/g). 10:2 fluorotelomer sulfonic acid (10:2 FTS) appeared in only two samples but exhibited the highest concentrations among the fluorotelomer sulfonates (1.15 and 3.31 ng/g). While 7:3 fluorotelomer carboxylic acid (7:3 FTCA) was detected in 28 samples at relatively high concentrations (median: 0.23 ng/g; IQR: 0.17–0.31 ng/g), the shorter-chain oxidative transformation products of fluorotelomer alcohols^56^ showed much lower detection rates, with 5:3 fluorotelomer carboxylic acid (5:3 FTCA) found in only one sample and 3:3 fluorotelomer carboxylic acid (3:3 FTCA) not detected in any samples. 5:3 FTCA has an estimated human serum elimination half-life of ∼43 days,^57^ comparable to PFHxA and PFHpA,^57^ which were also rarely detected here (*n* = 4 and *n* = 2, respectively).

### 3.2 Temporal Changes and Mixture Composition

PFAS concentrations in the human liver declined markedly over the 24-year study period, consistent with the global phaseout of the most widely produced legacy compounds (Figure 2). A weighted linear regression of log-transformed total PFAS (∑PFAS) concentrations against specimen collection year showed a significant decreasing trend (*p* < 0.0001; Figure 2). The modeled geometric mean ∑PFAS concentration decreased from 13 ng/g in 2000 to 0.74 ng/g in 2024, corresponding to a 94% reduction over the full sampling period.

**Figure 2:**
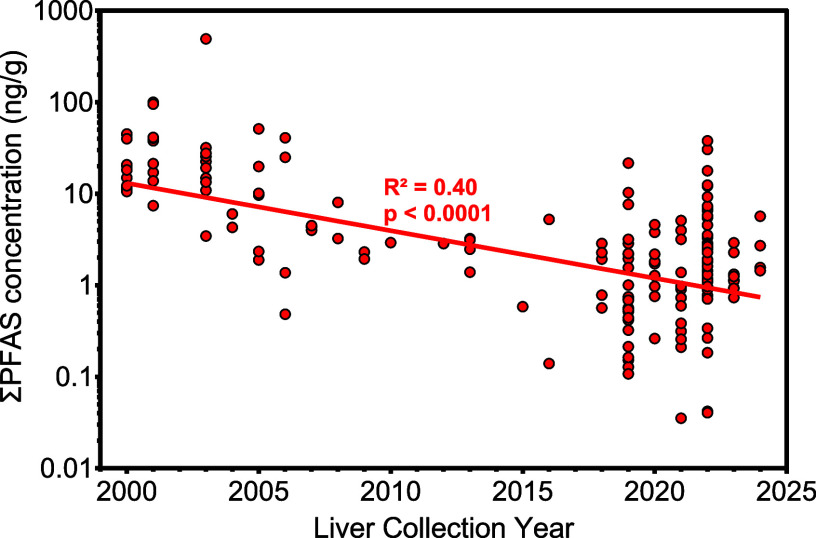
Temporal trends in the total concentration of PFAS in human liver samples collected from 2000 to 2024. Each point represents an individual liver sample with an exact sampling year recorded. The *y*-axis represents the sum of PFAS detected per sample (ng/g frozen weight) on a logarithmic scale. A weighted linear regression was performed on log_10_-transformed sum PFAS concentrations, with each data point weighted by the inverse of the number of samples collected in that year.

While the overall average concentration declined over time, ∑PFAS concentrations remained highly variable, spanning nearly 4 orders of magnitude (0.04 to 492 ng/g). Decade-level variability may reflect occupational exposures or proximity to highly contaminated drinking water. However, donor residential and occupational histories were unavailable, and biobank location does not necessarily reflect donor geography. Therefore, geographic confounding of temporal trends cannot be fully ruled out, although the consistency of declines across multiple phased-out PFAS classes supports a genuine temporal effect.

In the early 2000s, ∑PFAS levels frequently exceeded 20 ng/g, with 16 samples >40 ng/g and one reaching 492 ng/g in 2003. From 2020 to 2024, 36 samples exceeded 2 ng/g and 17 exceeded 5 ng/g, with a maximum of 38 ng/g in 2022. Decade-level comparisons further illustrate this shift: median ∑PFAS levels were 18 ng/g (IQR: 11–35 ng/g) in the early 2000s (2000–2005), 3.6 ng/g (IQR: 2.2–5.4 ng/g) in the late 2000s (2006–2010), and 1.7 ng/g (IQR: 0.93–3.4 ng/g) from 2020 onward. However, interindividual variability within single years remained substantial. In 2003, concentrations ranged 144-fold (3.4–492 ng/g), and in 2022, they spanned nearly 950-fold (0.04–38 ng/g), exceeding the magnitude of decade-level changes. While regulatory restrictions have reduced overall liver burdens, these data underscore ongoing exposures that are not declining in certain subsets of the population, warranting further investigation. Interestingly, weighted linear regressions of log-transformed concentrations for individual PFAS against collection year revealed compound-specific trends (Figures S2 and S5), with significant declines for several legacy sulfonates and carboxylates (PFOS, PFOA, PFDoA) but no change for others (PFHxS, FOSA, N-MeFOSAA) and a significant decrease for 8:2 FTS. To evaluate whether this trend reflects true temporal changes rather than shifts in the sampled population, multivariate regression models were used to adjust for age, sex, and liver health status (see section below).

The relative composition of PFAS in human livers shifted markedly over the 24-year study period (Figure 3). In the early 2000s, perfluoroalkyl sulfonic acids (PFSA) dominated, comprising >85% of ∑PFAS on average in 2000–2004 (e.g., 94% in 2003; 78% in 2006). During this period, average PFSA concentrations reached 33 ng/g (2001), with sulfonamides contributing up to 3.9 ng/g and fluorotelomer sulfonates <2 ng/g. From 2010 onward, PFSA levels declined sharply (e.g., 1.02 ng/g in 2023 vs 19–33 ng/g in 2000–2001), while fluorotelomers and sulfonamides emerged as larger mixture components, with fluorotelomers accounting for ∼50% of ∑PFAS (1.4 ng/g) by 2023, and sulfonamides contributing 2.8 ng/g (32% of total) in 2022.

**Figure 3:**
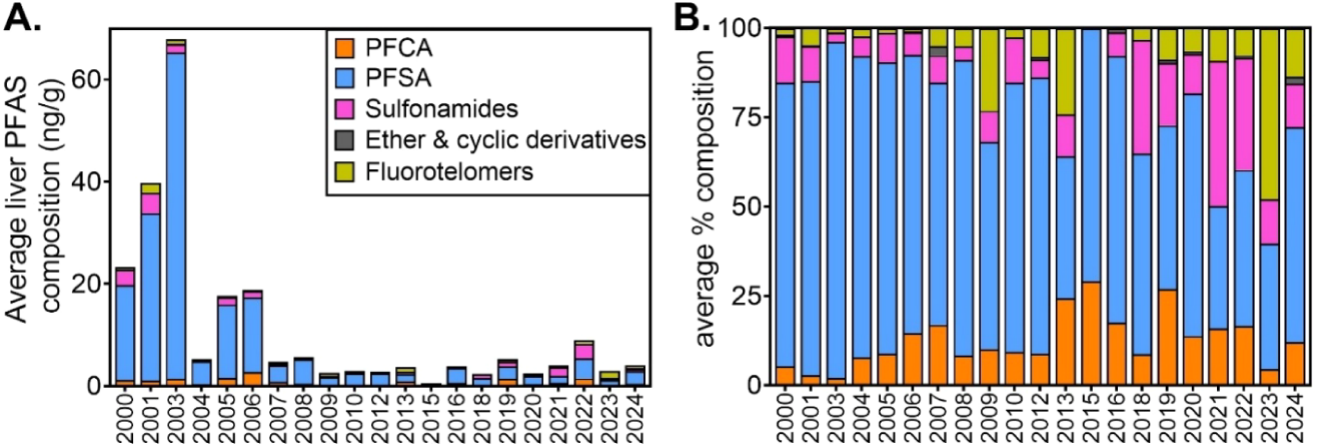
Temporal trends in the chemical class composition of PFAS in human liver tissue. Panel A shows the average concentrations (ng/g) of each PFAS class in liver tissue, calculated as the weighted mean for each year based on available samples. Panel B shows the relative composition (%), derived by normalizing the concentrations within each year to sum to 100%, highlighting class-specific shifts in PFAS profiles across the 24-year study period. PFAS were grouped into five classes: perfluoroalkyl carboxylic acids (PFCA), perfluoroalkyl sulfonic acids (PFSA), sulfonamides and precursors, chlorinated ether and cyclic PFAS derivatives, and fluorotelomer-based PFAS.

PFCA were a minor component in the early years (<8% of total PFAS in 2000–2004), but peaked in both relative and absolute terms in 2006 (2.7 ng/g, 15%) and again in 2019 (1.4 ng/g, 27%). Ethers and cyclic derivatives, such as 9ClPF3ONS, 3,6-OPFHpA, and PFECHS, were consistently rare yet detectable from 2000 onward, with the highest concentration (0.13 ng/g) in 2007. Our data demonstrate a compositional shift from sulfonate-dominated profiles in the early 2000s to increasingly diverse mixtures characterized by growing sulfonamide and fluorotelomer contributions. The persistence of legacy sulfonates alongside emerging chemistries underscores the need for continued monitoring of PFAS use patterns, environmental releases, and bioaccumulation in human tissues. These trends reflect changing exposure pathways and the widespread adoption of replacement chemistries.^53^

### 3.3 Role of Age, Sex, Liver Health, and Sampling Year

Significant associations (*p* < 0.05) were observed in the MLR models for all PFAS with at least one predictor in the 205-sample subset with complete metadata for all predictors: sampling year, sex, liver health status, and donor age (Figure 4). The sampling year, included as a fixed effect alongside the other covariates, was the most consistent determinant, associated with 13 of 15 compounds and with ∑PFAS. The largest decade-scale declines were observed for 8:2 FTS (−44%) and L-PFOS (−39%) (*p* < 0.01, Figure 4B). Significant decreases also occurred for Br-PFOS (−38%), L-N-MeFOSAA (−37%), PFDS (−35%), PFOA (−24%), L-N-EtFOSAA (−18%), FOSA (−12%), Br-N-MeFOSAA (−11%), PFNA (−9.3%), PFUdA (−6.3%), and PFDA (−4.8%) (*p* < 0.05). In contrast, L-PFHxS (−1.7%) and PFDoA (−0.38%) showed nonsignificant temporal changes. Interestingly, 9ClPF3ONS was associated with a 7% decade-scale increase (*p* < 0.01). The sampling year was also the most consistent determinant associated with the molecular weight range and the total number of perfluorinated carbons (η_*pfc*_) (Figure S6).

**Figure 4:**
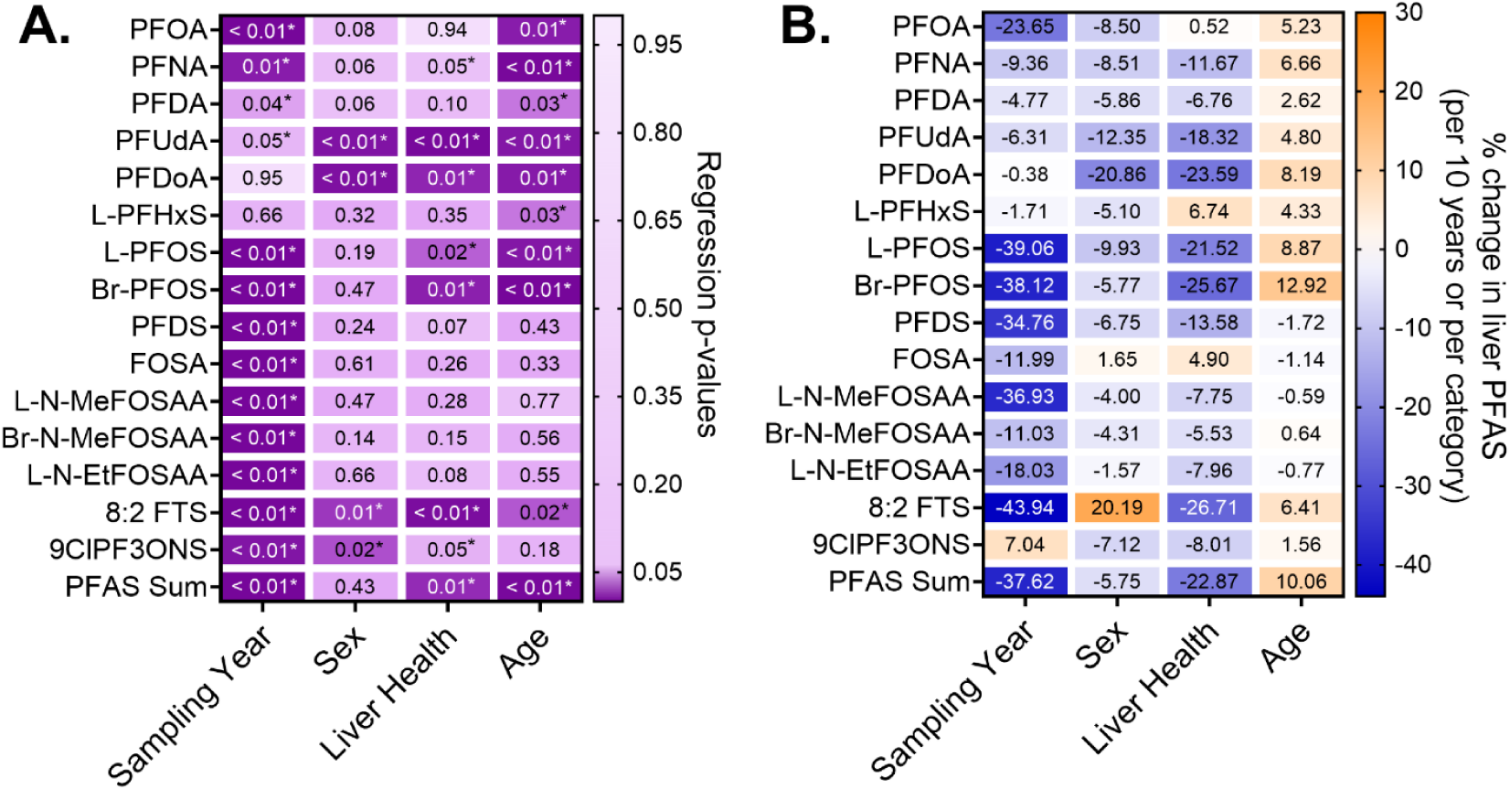
Results of multivariate linear regression analysis for PFAS detected in ≥30 of the 211 human liver samples collected from 2000 to 2024 (*n* = 205 with complete metadata). Panel A shows regression *p*-values (purple scale), with dark purple cells indicating statistical significance (*p* < 0.05, asterisk) and lighter purple tones representing higher *p*-values. Panel B shows regression β estimates transformed into percent change in liver PFAS concentrations per 10-year increase in sampling year or age, or per category shift in sex (male to female) or liver health status (healthy to NAFLD). Positive associations are shown in orange, and negative associations in blue. Models were based on log_10_-transformed PFAS concentrations; nondetects were replaced with MDL/2 prior to transformation.

Males exhibited significantly higher concentrations of long-chain compounds PFUdA (η_*pfc*_ = 11, +12%), PFDoA (η_*pfc*_ = 12, +21%), and 9ClPF3ONS (η_*pfc*_ = 8, +7.1%) compared to females (*p* < 0.05), while females showed higher levels of 8:2 FTS (+20%, *p* = 0.01). The male-biased presence of long-chain PFAS aligns with human biomonitoring studies attributing enhanced PFAS elimination in females to menstruation, childbirth, and lactation.^42^ Most female donors were postmenopausal (90 of 106 aged 46–85), and the loss of menstrual elimination may have contributed to higher concentrations of select PFAS in females. Sex-specific renal transporter expression, particularly organic anion transporters involved in PFAS excretion, has been proposed as a mechanism in rodent studies.^25^ However, unlike in rodents,^58^ renal transporters involved in PFAS clearance are not differentially expressed between sexes in humans.^59^ Consistently, the remaining PFAS and ∑PFAS showed no significant sex differences (Figure 4), aligning with human exposure studies that report no consistent sex effects on PFAS half-lives.^19,60^

Liver health status significantly influenced PFAS levels, with NAFLD associated with lower concentrations across 7 of 15 compounds and ∑PFAS: PFNA (−12%), PFUdA (−18%), PFDoA (−24%), L-PFOS (−22%), Br-PFOS (−26%), 8:2 FTS (−27%), 9ClPF3ONS (−8%) and ∑PFAS (−23%). Although liver health status and sampling year were correlated (*R* ^2^ = 0.43) and consistent with a greater proportion of NAFLD samples being collected in more recent years, multivariate analysis confirmed independent effects of both variables. Interestingly, NAFLD was associated with lower concentrations of PFAS with a molecular weight range of 500–600 and/or >600 g/mol, and an η_*pfc*_ of 8, 10, and 11 (Figure S6). This observation is consistent with studies measuring PFOS, PFHxS, PFOA, and PFNA in liver specimens from cirrhotic patients,^61^ as well as reduced liver PFOS and PFNA concentrations in mice fed a high-fat diet.^62,63^ The observed directionality is mechanistically plausible, as PFAS preferentially bind to albumin,^64^ phospholipids,^26,27,65^ and structural proteins^66^ rather than to neutral triglycerides that accumulate in steatotic liver,^43^ potentially reducing available binding sites for PFAS retention in steatotic liver tissue. Furthermore, OATP expression is frequently downregulated in fatty liver and alcoholic liver disease,^67^ which may further explain the negative associations between disease severity and hepatic PFAS concentrations.^68,69^ Because NAFLD develops over years, PFAS concentrations measured at the biopsy may not reflect binding conditions during disease progression. The gradual onset of steatosis alters liver composition and thus potentially PFAS partitioning well before clinical diagnosis. Dietary patterns associated with NAFLD, such as greater consumption of processed and packaged foods, may also independently influence PFAS exposure, while fiber intake may promote fecal PFAS elimination.^70^ However, dietary records were not available to evaluate these effects.

Age was positively associated with total PFAS burden (+10% per decade) and with individual compounds, including PFOA (+5.2%), PFNA (+6.7%), PFDA (+2.6%), PFUdA (+4.8%), PFDoA (+8.2%), L-PFHxS (+4.3%), L-PFOS (+8.9%), Br-PFOS (+13%), and 8:2 FTS (+6.4%) (all *p* < 0.05). Overall, similar trends were also observed with respect to PFAS molecular weight and the number of perfluorinated carbons (Figure S6). These associations align with the long elimination half-lives of these compounds,^19,71^ enabling gradual bioaccumulation, and may be further compounded by age-dependent decreases in PFAS excretion rates.^72,73^ Notably, PFDS, a long-chain PFSA with strong protein and lipid-binding affinity,^64,65^ showed no age correlation, while sulfonamide precursors (FOSA, L-N-MeFOSAA) were similarly age-independent, possibly reflecting their metabolic transformation to PFOS rather than long-term hepatic accumulation.^74^

### 3.4 Extractable Organofluorine in Human Liver Largely Unexplained by Targeted PFAS

Results from this study show large declines in targeted PFAS across years in human liver samples; however, preliminary data on EOF concentrations in eight of these samples suggest that only a small fraction of organofluorine in the liver is captured by the targeted analytes (Figure 5). On average, EOF concentrations were ∼13-fold higher than the fluorine-equivalent concentrations of targeted PFAS (14 ± 8.9 ng F/g; range: 7.1–29 ng F/g). This discrepancy was consistent in the matched samples: targeted PFAS accounted for only ≈8.5% and ≈3.9% of the total EOF in Donors 1 and 2, respectively.

**Figure 5:**
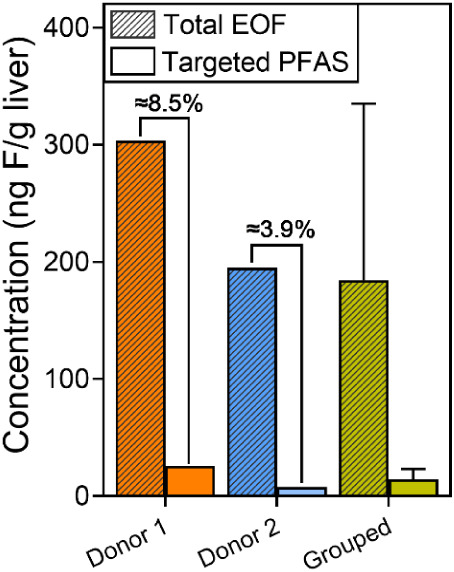
Comparison of extractable organofluorine (EOF) and targeted PFAS in human liver. Donors 1 and 2 were directly analyzed for EOF and targeted PFAS. Grouped data (“Grouped”) represent eight livers measured for EOF and 11 livers measured for targeted PFAS, matched by donor age, sex, sampling year, and biobank location. Percentages above bars indicate the fraction of EOF explained by targeted PFAS.

Mass balance analysis of pooled serum samples from the Tromsø Cohort (1986–2015) showed that 12 targeted PFAS accounted for ∼54% of EOF in 1986, ∼90% in 2007, and ∼63% in 2015.^75^ Subsequent suspect screening of the same pools attributed up to 63% of EOF in 2015 to fluorinated pharmaceuticals, substantially closing the mass balance in more recent samples, while a considerable fraction of EOF remained unidentified in earlier years.^76^ The higher proportion of unidentified EOF observed in liver tissue here may therefore reflect the presence of organofluorine compounds beyond the targeted PFAS, particularly fluorinated pharmaceuticals. Examples of highly prescribed fluorinated drugs introduced in the early 2000s include tyrosine kinase inhibitors (e.g., sorafenib), statins (e.g., rosuvastatin), antipsychotics (e.g., aripiprazole), and selective serotonin reuptake inhibitors (e.g., escitalopram).^77^ While not all of these are PFAS by definition, they or their metabolites all contribute to total hepatic organofluorine detectable by EOF. Fluorinated agrochemicals also represent a potential source of environmental organofluorine, with CF_3_-substituted compounds such as fipronil linked to hepatic toxicity.^78^ Widespread use of fluorinated pharmaceuticals and agrochemicals contributes to elevated organofluorine burdens in wastewater effluents and biosolids.^79^ Together with our EOF data, these findings underscore the growing burden of organofluorine contamination in ecosystems and humans.

### 3.5 Implications for PFAS Exposures and Adverse Health Effects

These results reveal substantial hepatic concentrations of both legacy and emerging PFAS. Despite two decades of regulatory action, individuals sampled as recently as 2022 exhibited liver ∑PFAS burdens exceeding 30 ng/g, increasingly driven by weakly regulated compounds like fluorotelomers, sulfonamides, and unregulated replacement chemicals like 9ClPF3ONS. Nevertheless, ∑PFAS concentrations (largely driven by PFOS) declined by 94% when adjusted by sampling year in the weighted linear regression model and by 68% after adjusting for donor demographics in the multivariate linear regression models, highlighting that donor demographics uniquely contribute to specimen concentration interindividual variability. Declines in phased-out compounds (PFOS, PFOA) indicate regulatory effectiveness, yet substitution with replacement chemistries sustains exposure and introduces compounds with limited toxicological characterization. Age, sex, and liver health each influenced hepatic burdens, underscoring the need to incorporate physiological variability into toxicokinetic models for predicting hepatic toxicity risks. Given the complex, shifting mixtures observed, with declining legacy compounds but emerging replacements, single-chemical regulatory approaches may be insufficient. Instead, structure-based regulatory frameworks that consider chain length, degree of fluorination, and potential for biotransformation to persistent and/or toxic metabolites could more effectively address the evolving landscape of PFAS exposure and associated risks.

Beyond targeted PFAS, the EOF analysis of selected livers revealed that >90% of extractable organofluorine remained unidentified, suggesting substantial hepatic levels of nontargeted PFAS, fluorinated pharmaceuticals, and pesticides. Resolving the hepatic organofluorine mass balance requires future studies to systematically measure total EOF and analyze known fluorinated pharmaceuticals and agrochemicals from the same liver tissue. EOF analysis of more recently collected livers is expected to reveal an even greater discrepancy, given the increasing production and use of novel fluorinated compounds across industrial, pharmaceutical, and agricultural sectors. Access to donor medical records would enable identification of the dominant pharmaceutical classes potentially driving hepatic organofluorine exposure.

Our reported liver concentration data can be contextualized against published effect levels to assess potential hepatotoxic risks through two approaches. First, measured concentrations (ng/g) can be converted to estimated free liver concentrations for comparison with *in vitro* hepatic end points (e.g., steatosis).^62,80^ Second, rodent points of departure can be translated to human liver-equivalent concentrations using physiologically based toxicokinetic (PBTK) modeling.^81,82^ This screening framework determines whether peak observed concentrations approach biologically relevant effect ranges and can be extended to mixture assessments using hazard index approaches.^83^

Future research priorities include (1) quantifying protein- and lipid-bound PFAS across organs to identify tissue components that drive PFAS accumulation in target organs (e.g., phospholipids and structural proteins), (2) integrating physiological factors into PBTK models to validate the demographic and health-related determinants of PFAS accumulation identified here, (3) developing liver-specific toxicity benchmarks for emerging PFAS using *in vitro* and rodent data, (4) implementing PFAS class- and structure-based regulatory frameworks that address complex mixtures, transformable precursors, and replacement chemicals, and (5) characterizing and quantifying unidentified EOF in the human liver (e.g., fluorinated pharmaceuticals and agrochemicals). The persistent hepatic accumulation of both legacy and replacement PFAS in this large human cohort demonstrates the urgent need to expand regulatory oversight beyond currently controlled substances.

## Supplementary Material



## Notes

PFAS compounds of interest, LC-MS/MS conditions, specimen demographics, ENVI-Carb solid phase extraction (SPE) methodology, LC-MS/MS internal standard recoveries and QC/QA thresholds, PFAS method detection limits, extractable organofluorine analysis QC/QA, EOF method detection limits, EOF and targeted PFAS analysis donor specimen demographics, weighted linear regression plots, and multivariate linear regression analysis (PDF)
