# Supplementary material for: Presence of Legacy and Emerging PFAS in Human Liver Specimens Banked in the United States from 2000 to 2024
Source: Environ Sci Technol. 2026 Apr 7;60(15):11281–92. doi: 10.1021/acs.est.5c14780 (PMC13104034; doi:10.1021/acs.est.5c14780)
Supplement: Supplementary file 1 [file es5c14780_si_001.pdf]

Supporting Information

Presence of Legacy and Emerging PFAS in Human Liver Specimens Banked in the United States from 2000-2024

Juliana Agudelo Areiza[1], Jitka Becanova[2], Simon Vojta[2], Johanna Ganglbauer[2], Udayan Apte[3], Luigi Brunetti[4], Sean Kumer[5], Faiz Haque[6,7], Euna Kim[6], Elsie M. Sunderland[6], Rainer Lohmann[2], Fabian C. Fischer[1,], Angela Slitt[1,*]*

[1] Department of Biomedical and Pharmaceutical Sciences, College of Pharmacy, University of Rhode Island, Kingston, Rhode Island 02881, United States

[2] Graduate School of Oceanography, University of Rhode Island, Narragansett, Rhode Island 02881, United States

[3] Department of Pharmacology, Toxicology and Therapeutics, University of Kansas Medical Center, Kansas City, Kansas 66103, United States

[4] Pharmacy Practice and Administration, Ernest Mario School of Pharmacy, Rutgers University, Piscataway, New Jersey, 08854.

[5] Department of Surgery, University of Kansas Medical Center, Kansas City, Kansas 66103, United States

[6] Harvard School of Engineering and Applied Sciences, Harvard University, Boston, Massachusetts 02115, United States

[7] University of Vienna, Wien, Austria

***Address correspondence to: angela_slitt@uri.edu, fabian.fischer@uri.edu**

<u>Content overview:</u> 19 Pages, 9 Tables, and 6 Figures.

Section	Contents	Page
S-1.	PFAS Compounds of Interest.	S3
S-2.	LC-MS/MS Conditions.	S4
S-3	Specimen Demographics.	S6
S-4	ENVI-Carb™ Solid Phase Extraction (SPE) Methodology.	S7
S-5.	LC-MS/MS Internal Standard Recoveries and QC/QA Thresholds.	S7
S-6.	PFAS Method Detection Limits.	S11
S-7.	Extractable Organofluorine Analysis QC/QA.	S13
S-8.	EOF Method Detection Limits.	S13
S-9.	EOF and Targeted PFAS Analysis Donor Specimen Demographics.	S14
S-10.	Weighted Linear Regression Plots.	S15
S-11.	Multivariate Linear Regression Analysis.	S19

Section S1. PFAS Compounds of Interest.

Table S1. List of PFAS analytes measured. PFAS acronym, compound full name, and CAS number are presented herein.

Acronym	Compound Name	CAS Number
PFPrA	Perfluoropropanoic acid	422-64-0
PFBA	Perfluorobutanoic acid	375-22-4
PFPeA	Perfluoropentanoic acid	2706-90-3
PFHxA	Perfluorohexanoic acid	307-24-4
PFHpA	Perfluoroheptanoic acid	375-85-9
PFOA	Perfluorooctanoic acid	335-67-1
PFNA	Perfluorononanoic acid	375-95-1
PFDA	Perfluorodecanoic acid	335-76-2
PFUdA	Perfluoroundecanoic acid	2058-94-8
PFDoA	Perfluorododecanoic acid	307-55-1
PFTrDA	Perfluorotridecanoic acid	72629-94-8
PFTeDA	Perfluorotetradecanoic acid	376-06-7
3:3 FTCA (FPrPA)	2H,2H,3H,3H-Perfluorohexanoic acid	356-02-5
5:3 FTCA (FPePA)	2H,2H,3H,3H-Perfluorooctanoic acid	914637-49-3
7:3 FTCA (FHpPA)	2H,2H,3H,3H-Perfluorodecanoic acid	812-70-4
8:2 FTCA (FOEA)	8:2 Perfluorocarboxylic acid	27854-31-5
PFPrS	Perfluropropanesulfonic acid	423-41-6
PFBS	Perfluorobutanesulfonic acid	375-73-5
PFPeS	Perfluoropentanesulfonic acid	2706-91-4
L-PFHxS	Perfluorohexanesulfonic acid	355-46-4
Br-PFHxS	Perfluorohexanesulfonic acid	355-46-4
PFHpS	Perfluroheptanesulfonic acid	375-92-8
L-PFOS	Perfluorooctanesulfonic acid	1763-23-1
Br-PFOS	Perfluorooctanesulfonic acid	1763-23-1
PFNS	Perfluorononanesulfonic acid	68259-12-1
PFDS	Perfluorodecanesulfonic acid	335-77-3
MeFBSA	N-Methylperfluorobutane sulfonamide	68298-12-4
N-EtFBSA-M	N-Ethylperfluorobutane sulfonamide	40630-67-9
FBSA	Perfluorobutyl sulfonamide	30334-69-1
FPeSA	Perfluoropentane sulfonamide	82765-76-2
FHxSA	Perfluorohexane sulfonamide	41997-13-1
FHpSA	Perfluoroheptane sulfonamide	82765-77-3
FOSA	Perfluorooctane sulfonamide	754-91-6
L-N-MeFOSAA	N-methyl perfluorooctanesulfonamidoacetic acid	2355-31-9
Br-N-MeFOSAA	N-methyl perfluorooctanesulfonamidoacetic acid	2355-31-9
L-N-EtFOSAA	N-ethyl perfluorooctanesulfonamidoacetic acid	2991-50-6
Br-N-EtFOSAA	N-ethyl perfluorooctanesulfonamidoacetic acid	2991-50-6
MeFOSA	N-Methylperfluorooctane sulfonamide	31506-32-8
EtFOSA	N-Ethylperfluorooctane sulfonamide	4151-50-2
HFPO-DA (Gen X)	Hexafluoropropylene oxide dimer acid	13252-13-6
DONA (ADONA)	4,8-Dioxa-3H-perfluorononanoic acid	919005-14-4
9ClPF3ONS	9-Chlorohexadecafluoro-3-oxanonane-1-sulfonic acid	756426-58-1

11ClPF3OUdS	11-Chloroeicosafluoro-3-oxaundecane-1-sulfonic acid	763051-92-9
3,6-OPFHpA	Nonafluoro-3,6-dioxaheptanoic acid	151772-58-6
PF4OPeA	Perfluoro-3-methoxypropanoic acid	377-73-1
PF5OHxA	Perfluoro-4-methoxybutanoic acid	863090-89-5
PFECHS	Perfluoroethylcyclohexanesulfonic acid	646-83-3
PFEESA	Perfluoro(2-ethoxyethane)sulfonic acid	113507-82-7
N-AP-FHxSA	N-[3-(Dimethylamino)propyl] Perfluorohexane sulfonamide	50598-28-2
N-TAmP-FHxSA	N-[3-(Trimethylamino)propyl] Perfluorohexane sulfonamide	38850-51-0
4:2 FTS	1H,1H,2H,2H-Perfluorohexanesulfonic acid	757124-72-4
6:2 FTS	1H,1H,2H,2H-Perfluorooctanesulfonic acid	27619-97-2
8:2 FTS	1H,1H,2H,2H-Perfluorodecanesulfonic acid	39108-34-4
10:2 FTS	1H,1H,2H,2H-Perfluorododecanesulfonic acid	120226-60-0

Section S2. LC-MS/MS Conditions.

Table S2. LC-MS/MS conditions. Instrumental conditions, including target analyte set, analyte precursor mass (Da), fragment mass (Da), declustering potential (DP), collision energy (CE), retention time (RT), and PFAS mass-labeled internal standards, are presented herein. EIS represents the extractable internal standard, and NIS is the non-extractable internal standard.

Acronym	Target analyte set	Precursor Mass (Da)	Fragment Mass (Da)	DP (V)	CE (V)	RT (min)	Internal Standard (IS)
PFBA	Core	212.98	168.989	-25	-12	1.6	13C4_PFBA
PFPeA	Core	262.98	218.9858	-20	-12	3.5	13C5_PFPeA
PFHxA	Core	312.97	268.9824	-25	-12	6.2	13C5_PFHxA
PFHpA	Core	362.97	318.9791	-25	-12	7.3	13C4_PFHpA
PFOA	Core	412.97	368.9761	-25	-14	8	13C8_PFOA
PFNA	Core	462.96	418.9732	-25	-14	8.5	13C9_PFNA
PFDA	Core	512.96	468.97	-25	-16	8.84	13C6_PFDA
PFUdA	Core	562.96	518.9673	-25	-18	9.2	13C7_PFUdA
PFDoA	Core	612.95	568.9634	-25	-18	9.45	13C2_PFDoA
PFTrDA	Core	662.95	618.9617	-30	-19	9.7	13C2_PFDoA
PFTeDA	Core	712.95	668.9579	-30	-22	9.9	13C2_PFTeDA
PFBS	Core	298.94	79.9572	-55	-58	4.3	13C3_PFBS
PFPeS	Core	348.94	79.9572	-60	-66	6.5	13C3_PFBS
L-PFHxS	Core	398.94	79.9572	-60	-74	7.4	13C3_PFHxS
Br-PFHxS	Core	398.94	79.9572	-60	-74	7.22	13C3_PFHxS
PFHpS	Core	448.93	79.9572	-65	-88	8.1	13C3_PFHxS
L-PFOS	Core	498.93	79.9573	-65	-108	8.5	13C8_PFOS
Br-PFOS	Core	498.93	79.9573	-65	-108	8.34	13C8_PFOS
PFNS	Core	548.93	79.9571	-50	-120	8.9	13C8_PFOS
PFDS	Core	598.92	79.9569	-45	-120	9.25	13C8_PFOS
FBSA	Core	297.96	77.9653	-40	-50	5.3	13C8_FOSA
FPeSA	Core	347.96	77.9653	-60	-35	7.2	13C8_FOSA
FHxSA	Core	397.95	77.9654	-10	-70	8	13C8_FOSA
FHpSA	Core	447.96	77.9653	-60	-35	8.5	13C8_FOSA
FOSA	Core	497.94	77.9654	-60	-85	9	13C8_FOSA
4:2 FTS	Core	326.97	306.9676	-50	-28	6.1	13C2 4:2 FTS

6:2 FTS	Core	426.97	406.9615	-50	-32	8	13C2 6:2 FTS
8:2 FTS	Core	526.96	506.9557	-50	-40	8.9	13C2 8:2 FTS
10:2 FTS	Core	626.95	606.9479	-50	-40	9.5	13C2 8:2 FTS
PFPrA	Extended	163.08	118.9919	-25	-12	1	13C4_PFBA
3:3 FTCA (FPrPA)	Extended	241	136.9892	-20	-20	3.2	13C5_PFPeA
5:3 FTCA (FPePA)	Extended	341	236.9892	-20	-20	7.4	13C4_PFHpA
7:3 FTCA (FHpPA)	Extended	441	336.9892	-70	-18	8.6	13C9_PFNA
8:2 FTCA (FOEA)	Extended	476.98	392.9767	-50	-20	8.6	13C9_PFNA
PFPrS	Extended	248.95	79.9572	-55	-58	1.7	13C3_PFBS
MeFBSA	Extended	311.97	218.9862	-40	-25	7.5	13C8_FOSA
N-EtFBSA-M	Extended	325.99	218.9862	-30	-20	8	13C8_FOSA
L-N-MeFOSAA	Extended	569.97	418.9734	-75	-28	9.15	d3-MeFOSAA
Br-N-MeFOSAA	Extended	569.97	418.9734	-75	-28	9.05	d3-MeFOSAA
L-N-EtFOSAA	Extended	583.98	418.9731	-50	-36	9.25	d5-EtFOSAA
Br-N-EtFOSAA	Extended	583.98	418.9731	-50	-36	9.25	d5-EtFOSAA
MeFOSA	Extended	511.96	168.9889	-95	-36	9.5	d-MeFOSA
EtFOSA	Extended	525.98	168.989	-90	-36	9.6	d-EtFOSA
HFPO-DA (Gen X)	Extended	284.98	168.9889	-30	-12	6.75	13C3_HFPO-DA
DONA (ADONA)	Extended	376.97	250.9754	-55	-16	7.5	13C4_PFHpA
9ClPF3ONS	Extended	530.89	350.945	-120	-30	8.8	13C8_PFOS
11ClPF3OUdS	Extended	630.89	450.9383	-160	-40	9.4	13C8_PFOS
3,6-OPFHpA	Extended	200.98	84.9907	-37	-18	5.9	13C8_PFOS
PF4OPeA	Extended	228.97	84.9907	-21	-21	2	13C8_PFOS
PF5OHxA	Extended	278.97	84.9907	-34	-28	4.3	13C8_PFOS
PFECHS	Extended	460.93	98.9557	-10	-70	8	13C3_PFHxS
PFEESA	Extended	314.94	134.9875	-150	-30	5.5	13C3_PFHxS
N-AP-FHxSA	Extended	483.04	168.9898	-30	-34	7.84	13C3_PFHxS
N-TAmP-FHxSA	Extended	497.06	168.9898	-40	-34	7.2	13C3_PFHxS
13C3_PFBA		215.99	171.9994	-25	-12	1.6	NIS
13C2_PFHxA		314.99	272.9961	-25	-12	6.3	NIS
13C4_PFOA		416.99	371.9867	-25	-14	8	NIS
13C5_PFNA		467.99	422.9898	-25	-14	8.5	NIS
13C2_PFDA		514.98	469.9736	-25	-16	8.81	NIS
18O2_PFHxS		402.95	102.9643	-60	-50	7.4	NIS
13C4_PFOS		502.94	79.9574	-65	-108	8.5	NIS
13C4_PFHpA		366.98	321.99	-25	-12	7.3	EIS
13C8_PFOA		420.99	376.0003	-25	-14	8	EIS
13C9_PFNA		471.99	427.0009	-25	-14	8.5	EIS
13C4_PFBA		216.99	171.9989	-25	-12	1.6	EIS
13C5_PFPeA		267.99	222.9995	-20	-12	3.5	EIS
13C5_PFHxA		317.99	272.9961	-25	-12	6.3	EIS

13C6_PFDA		518.98	473.9867	-25	-16	8.81	EIS
13C7_PFUdA		569.98	524.9864	-25	-18	9.16	EIS
13C2_PFDoA		614.96	569.9673	-25	-18	9.45	EIS
13C2_PFTeD A		714.96	669.963	-55	-25	9.9	EIS
13C3_PFBS		301.95	79.9574	-55	-58	4.4	EIS
13C3_PFHxS		401.95	79.9572	-60	-50	7.4	EIS
13C8_PFOS		506.96	79.9572	-65	-108	8.5	EIS
13C8_FOSA		505.97	77.9654	-60	-85	9	EIS
d3-MeFOSAA		572.98	418.9744	-75	-28	9.15	EIS
d5-EtFOSAA		589.01	418.9727	-75	-37	9.25	EIS
d-MeFOSA		514.98	168.9896	-90	-36	9.5	EIS
d-EtFOSA		531.01	168.9882	-90	-34	9.6	EIS
13C3_HFPO-DA		286.98	168.9889	-40	-10	6.75	EIS
13C2 4:2 FTS		328.98	80.9649	-50	-40	6.1	EIS
13C2 6:2 FTS		428.97	80.9651	-55	-60	8	EIS
13C2 8:2 FTS		528.97	80.9648	-55	-60	8.9	EIS

Section S3. Specimen Demographics

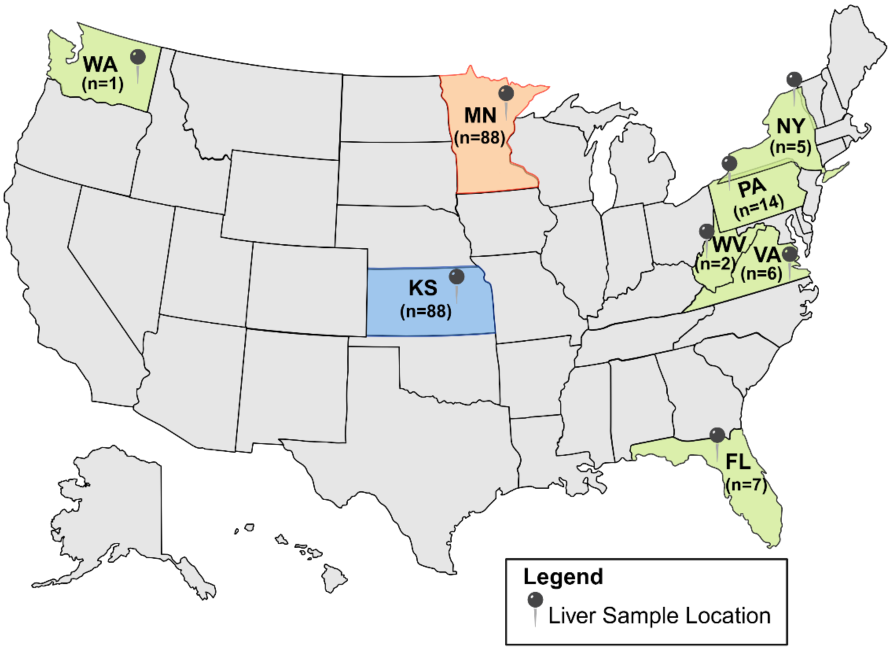

Figure S1. Map of human liver samples (n = sample size) location in selected U.S. states - Washington (WA), Kansas (KS), Minnesota (MN), New York (NY), Pennsylvania (PA), West Virginia (WV), Virginia (VA), and Florida (FL). Human liver samples were acquired from the following U.S. tissue banks: National Disease Research Interchange (NDRI) in green, the University of Kansas Medical Center (KUMC) in blue, and the University of Minnesota Liver Tissue Cell Distribution System (UMN LTCDS) in orange. Donor specimen locations were confirmed from the NDRI tissue bank, whereas specimens acquired from KUMC and UMN LTCDS were presumed to be from donors who resided near the respective tissue bank.

Section S4. ENVI-Carb™ Solid Phase Extraction (SPE) Methodology

Graphitized carbon solid phase extraction (SPE) was conducted using Superclean™ ENVI-Carb™ SPE cartridges (Millipore Sigma) for sample cleanup to remove any remaining sample biological matrix after PFAS extraction. Briefly, ENVI-Carb™ SPE cartridges (0.5 g/6 mL) were pre-cleaned on a manual 24-position MilliporeSigma Supelco Visiprep SPE Vacuum with 6 mL of 3% basic methanol and conditioned with 6 mL of LC/MS-grade methanol. Approximately 1.5 mL of sample was loaded onto the cartridge, and the pre-existing polypropylene tube was rinsed with ~500 µL of LC/MS-grade methanol to ensure quantitative transfer, and the rinse was combined with the sample twice. Next, an appropriately labeled pre-cleaned polypropylene tube was placed into the SPE test tube rack and placed under the corresponding SPE needle. The samples were allowed to flow through the cartridge at approximately 1 drop/second under vacuum control as needed.

Section S5. LC-MS/MS Internal Standard Recoveries and QC/QA Thresholds.

Table S3. Extractable internal standard (EIS) threshold (%) and recoveries (%) criteria for human liver PFAS LC-MS/MS data. Values outside of these ranges did not pass QC/QA and were not reported in the dataset.

Compound Name	Lower Threshold [%]	Recoveries [%]
IDA-13C4_PFBA	20	150
IDA-13C5_PFPeA	20	185
IDA-13C5_PFHxA	20	170
IDA-13C4_PFHpA	20	150
IDA-13C8_PFOA	20	150
IDA-13C9_PFNA	20	185
IDA-13C6_PFDA	20	150
IDA-13C7_PFUdA	20	180
IDA-13C2_PFDoA	20	180
IDA-13C2_PFTeDA	20	160
IDA-13C3_PFBS	20	190
IDA-13C3_PFHxS	20	175
IDA-13C8_PFOS	20	160
IDA-13C8_FOSA	20	180
IDA-d3-MeFOSAA	20	250
IDA-d5-EtFOSAA	20	235
IDA-d-MeFOSA	20	150
IDA-d-EtFOSA	20	150
IDA-13C3_HFPO-DA	20	185
IDA-13C2 4:2 FTS	20	300
IDA-13C2 6:2 FTS	20	300
IDA-13C2 8:2 FTS	20	365

Table S4. Extractable internal standard (EIS) recoveries (%) of experimental rat liver matrix spike controls for the human liver PFAS LC-MS/MS data. Values outside of the set EIS threshold (%) did not pass QC/QA and were not reported in the dataset.

Analytes	Matrix Spike 1	Matrix Spike 2	Matrix Spike 3	Matrix Spike 4	Matrix Spike 5	Matrix Spike 6	Matrix Spike 7	Matrix Spike 8	Matrix Spike 9	Matrix Spike 10	Matrix Spike 11	Matrix Spike 12	Matrix Spike 13	Matrix Spike 14	Matrix Spike 15	Matrix Spike 16	Matrix Spike 17	Matrix Spike 18	Matrix Spike 19	Matrix Spike 20	Matrix Spike 21	Matrix Spike 22	Matrix Spike 23	Matrix Spike 24
PFPrA	86.3	76.5	83.2	86.3	76.0	77.2	67.5	74.3	84.8	82.8	87.9	87.6	84.8	74.4	72.6	81.7	77.9	82.0	82.0	80.8	79.1	73.9	89.0	74.8
PFBA	89.1	82.1	84.3	87.3	76.1	80.2	71.4	75.2	85.9	91.6	85.5	85.7	83.8	75.9	71.9	80.8	79.1	81.5	84.3	76.9	82.0	70.4	85.4	73.9
PFPeA	99.6	115.1	130.8	124.4	94.7	116.6	111.5	87.7	129.9	77.4	128.4	106.7	135.3	104.0	95.7	112.8	101.1	116.8	98.5	88.4	123.8	101.7	135.1	105.6
PFHxA	94.7	79.3	88.4	106.0	111.3	108.3	72.4	81.8	81.6	98.7	88.5	106.2	92.1	87.8	74.1	82.2	68.5	85.2	85.1	91.7	103.1	76.0	89.6	79.8
PFHpA	86.0	82.8	98.7	106.3	109.6	103.4	68.9	81.3	82.4	70.9	78.3	101.8	89.2	96.5	76.0	75.7	67.3	83.8	95.9	85.4	97.7	69.6	84.2	72.9
PFOA_TOF MS	90.9	83.5	86.3	91.3	86.3	82.8	67.4	77.4	78.8	82.4	86.3	88.1	89.8	77.5	71.9	77.8	72.3	90.4	90.0	85.9	84.9	68.4	86.9	73.6
PFNA	80.6	65.0	60.5	84.4	76.4	74.2	60.5	77.3	81.2	73.0	96.6	95.3	80.0	78.8	67.0	81.0	76.5	72.0	94.5	67.3	85.6	65.6	86.2	73.3
PFDA	79.7	57.1	62.2	85.7	83.9	75.7	66.9	74.7	94.4	61.2	92.6	81.2	82.7	70.0	76.3	88.0	83.5	79.4	82.7	66.1	84.3	66.8	75.4	70.4
PFUdA	74.7	49.8	101.6	78.8	92.6	74.8	46.8	75.4	88.2	48.5	64.1	82.4	93.0	62.3	63.9	93.7	86.0	55.5	73.4	57.6	79.8	53.7	74.5	58.2
PFDoA	66.5	31.5	54.7	62.9	77.1	47.9	40.8	80.4	66.6	42.3	62.1	60.7	68.4	58.9	53.7	76.7	85.4	54.5	44.2	60.2	71.0	60.0	62.9	66.5
PFTrDA	66.5	31.5	54.7	62.9	77.1	47.9	40.8	80.4	66.6	42.3	62.1	60.7	68.4	58.9	53.7	76.7	85.4	54.5	44.2	60.2	71.0	60.0	62.9	66.5
PFTeDA	75.6	45.7	72.2	65.5	102.4	54.9	81.9	71.6	142.0	78.3	159.9	60.0	75.0	44.3	70.4	38.8	7.5	101.6	77.5	111.6	90.5	69.4	161.9	84.1
3:3 FTCA	105.7	117.5	129.5	131.7	100.5	132.3	120.2	96.1	132.4	102.2	143.4	124.1	141.5	117.6	109.5	118.9	106.5	126.6	108.0	106.6	137.1	116.4	152.1	118.3
5:3 FTCA	89.6	86.5	97.4	105.1	109.6	105.6	82.7	80.1	78.3	97.5	79.4	100.4	104.1	88.1	76.8	71.8	68.4	93.7	100.2	97.7	84.0	73.8	89.7	85.3
7:3 FTCA	76.9	58.7	71.7	85.6	84.4	82.8	67.0	80.6	82.2	70.8	90.5	93.7	102.5	68.4	56.5	78.4	76.9	70.9	96.2	72.1	73.5	65.3	95.9	66.7
FOEA	76.9	58.7	71.7	85.6	84.4	82.8	67.0	80.6	82.2	70.8	90.5	93.7	102.5	68.4	56.5	78.4	76.9	70.9	96.2	72.1	73.5	65.3	95.9	66.7
PFPrS	90.4	82.1	78.0	83.5	87.8	85.8	78.9	74.2	87.7	79.4	85.0	85.8	84.3	70.8	70.7	83.1	71.0	78.6	79.0	84.0	86.1	74.9	84.3	66.3
PFBS	94.2	84.0	77.3	83.0	93.7	103.7	75.3	81.3	93.2	53.1	91.5	93.4	91.1	69.7	85.0	86.9	75.9	96.4	82.6	89.5	89.2	79.7	94.9	71.8
PFPeS	94.2	84.0	77.3	83.0	93.7	103.7	75.3	81.3	93.2	53.1	91.5	93.4	91.1	69.7	85.0	86.9	75.9	96.4	82.6	89.5	89.2	79.7	94.9	71.8
L-PFHxS_TOF MS	95.1	95.5	90.0	95.3	102.7	103.2	74.2	79.3	94.2	58.4	97.3	92.4	103.7	81.0	84.8	85.7	75.1	103.5	92.8	88.2	82.4	79.0	98.4	82.0
Br-PFHxS_TOF MS	95.1	95.5	90.0	95.3	102.7	103.2	74.2	79.3	94.2	58.4	97.3	92.4	103.7	81.0	84.8	85.7	75.1	103.5	92.8	88.2	82.4	79.0	98.4	82.0
PFHpS	95.1	95.5	90.0	95.3	102.7	103.2	74.2	79.3	94.2	58.4	97.3	92.4	103.7	81.0	84.8	85.7	75.1	103.5	92.8	88.2	82.4	79.0	98.4	82.0
L-PFOS_TOF MS	86.7	85.0	84.6	87.2	81.4	78.2	67.3	70.3	76.4	78.3	87.9	81.0	91.7	75.9	68.2	72.4	68.0	88.3	88.8	81.2	77.1	68.5	85.7	76.5
Br-PFOS_TOF MS	86.7	85.0	84.6	87.2	81.4	78.2	67.3	70.3	76.4	78.3	87.9	81.0	91.7	75.9	68.2	72.4	68.0	88.3	88.8	81.2	77.1	68.5	85.7	76.5
PFNS	89.9	85.6	82.3	94.0	96.3	94.9	67.0	79.7	83.0	57.4	87.2	92.5	102.2	73.2	76.8	86.5	74.2	106.3	86.4	93.1	86.8	74.9	88.4	78.7
PFDS	89.9	85.6	82.3	94.0	96.3	94.9	67.0	79.7	83.0	57.4	87.2	92.5	102.2	73.2	76.8	86.5	74.2	106.3	86.4	93.1	86.8	74.9	88.4	78.7
MeFBSA	67.5	60.5	67.2	66.0	60.2	64.9	47.0	54.1	50.1	50.0	51.4	66.7	62.0	55.8	60.5	57.0	60.1	57.0	58.2	67.2	52.2	52.2	48.2	53.4
N-EtFBSA-M	67.5	60.5	67.2	66.0	60.2	64.9	47.0	54.1	50.1	50.0	51.4	66.7	62.0	55.8	60.5	57.0	60.1	57.0	58.2	67.2	52.2	52.2	48.2	53.4
FBSA	54.1	48.8	50.8	52.4	69.2	49.5	38.7	57.3	41.6	28.7	52.7	58.8	58.2	50.0	60.8	49.1	53.7	64.3	56.8	70.6	54.3	46.5	57.8	53.2
FPeSA	54.1	48.8	50.8	52.4	69.2	49.5	38.7	57.3	41.6	28.7	52.7	58.8	58.2	50.0	60.8	49.1	53.7	64.3	56.8	70.6	54.3	46.5	57.8	53.2
FHxSA	54.1	48.8	50.8	52.4	69.2	49.5	38.7	57.3	41.6	28.7	52.7	58.8	58.2	50.0	60.8	49.1	53.7	64.3	56.8	70.6	54.3	46.5	57.8	53.2
FHpSA	54.1	48.8	50.8	52.4	69.2	49.5	38.7	57.3	41.6	28.7	52.7	58.8	58.2	50.0	60.8	49.1	53.7	64.3	56.8	70.6	54.3	46.5	57.8	53.2
FOSA	54.1	48.8	50.8	52.4	69.2	49.5	38.7	57.3	41.6	28.7	52.7	58.8	58.2	50.0	60.8	49.1	53.7	64.3	56.8	70.6	54.3	46.5	57.8	53.2
L-N-MeFOSAA	97.5	109.1	91.2	83.2	51.4	91.5	71.8	80.1	98.1	64.8	76.8	79.0	103.9	88.1	77.7	96.0	64.9	71.7	79.7	64.7	83.5	81.7	66.3	92.9
Br-N-MeFOSAA	97.5	109.1	91.2	83.2	51.4	91.5	71.8	80.1	98.1	64.8	76.8	79.0	103.9	88.1	77.7	96.0	64.9	71.7	79.7	64.7	83.5	81.7	66.3	92.9
L-N-EtFOSAA	81.4	79.2	61.4	67.9	45.0	68.5	60.7	64.5	94.2	57.1	63.7	60.9	71.4	70.7	70.1	79.2	40.6	61.4	62.8	52.2	72.5	71.2	50.2	70.1
Br-N-EtFOSAA	81.4	79.2	61.4	67.9	45.0	68.5	60.7	64.5	94.2	57.1	63.7	60.9	71.4	70.7	70.1	79.2	40.6	61.4	62.8	52.2	72.5	71.2	50.2	70.1
MeFOSA	60.6	27.4	32.0	37.6	33.1	41.8	19.3	36.8	57.3	40.9	43.7	61.7	34.2	28.4	64.4	59.7	78.5	56.6	26.5	50.9	25.9	21.9	42.1	23.8
EtFOSA	58.4	27.0	31.5	27.5	28.6	40.7	21.8	37.7	61.3	34.8	46.6	57.4	28.1	28.7	70.7	45.1	52.9	48.3	21.7	39.2	25.1	17.6	44.2	25.7
HFPO-DA	99.9	69.6	82.8	75.7	91.2	88.5	88.3	88.9	94.5	87.0	95.4	101.3	84.5	62.0	70.7	87.1	86.2	75.3	77.3	87.0	90.8	76.9	100.9	68.1
DONA	89.6	86.5	97.4	105.1	109.6	105.6	82.7	80.1	78.3	97.5	79.4	100.4	104.1	88.1	76.8	71.8	68.4	93.7	100.2	97.7	84.0	73.8	89.7	85.3
9ClPF3ONS	93.1	89.5	92.5	96.7	89.1	84.1	77.7	75.6	87.8	82.2	81.8	89.8	94.6	78.1	75.1	85.9	71.9	78.6	92.1	89.0	85.5	76.4	78.7	74.0
11ClPF3OUdS	93.1	89.5	92.5	96.7	89.1	84.1	77.7	75.6	87.8	82.2	81.8	89.8	94.6	78.1	75.1	85.9	71.9	78.6	92.1	89.0	85.5	76.4	78.7	74.0
3,6-OPFHpA	93.1	89.5	92.5	96.7	89.1	84.1	77.7	75.6	87.8	82.2	81.8	89.8	94.6	78.1	75.1	85.9	71.9	78.6	92.1	89.0	85.5	76.4	78.7	74.0
PF4OPeA	93.1	89.5	92.5	96.7	89.1	84.1	77.7	75.6	87.8	82.2	81.8	89.8	94.6	78.1	75.1	85.9	71.9	78.6	92.1	89.0	85.5	76.4	78.7	74.0
PF5OHxA	93.1	89.5	92.5	96.7	89.1	84.1	77.7	75.6	87.8	82.2	81.8	89.8	94.6	78.1	75.1	85.9	71.9	78.6	92.1	89.0	85.5	76.4	78.7	74.0
PFECHS	95.3	96.7	94.0	100.5	90.2	95.8	79.5	75.4	90.5	83.7	89.2	91.0	98.2	84.7	75.6	82.6	71.0	84.7	92.9	91.7	82.5	74.6	88.2	75.7
PFEESA	95.3	96.7	94.0	100.5	90.2	95.8	79.5	75.4	90.5	83.7	89.2	91.0	98.2	84.7	75.6	82.6	71.0	84.7	92.9	91.7	82.5	74.6	88.2	75.7
N-AP-FHxSA	95.3	96.7	94.0	100.5	90.2	95.8	79.5	75.4	90.5	83.7	89.2	91.0	98.2	84.7	75.6	82.6	71.0	84.7	92.9	91.7	82.5	74.6	88.2	75.7
N-TAmP-FHxSA	95.3	96.7	94.0	100.5	90.2	95.8	79.5	75.4	90.5	83.7	89.2	91.0	98.2	84.7	75.6	82.6	71.0	84.7	92.9	91.7	82.5	74.6	88.2	75.7
4:2 FTS	88.5	258.6	123.5	126.8	103.0	152.2	186.1	100.6	104.9	104.4	298.1	102.7	257.6	192.2	104.5	98.2	78.9	199.6	78.0	87.0	187.1	143.7	274.7	219.8
6:2 FTS	88.4	173.8	105.1	105.7	82.2	118.1	118.0	100.8	78.7	38.0	184.2	91.9	194.8	137.8	92.0	70.1	60.8	137.3	73.4	68.2	123.2	109.9	157.8	169.2
8:2 FTS	68.6	115.4	60.4	72.0	56.9	80.6	61.3	79.0	77.2	44.2	120.3	67.8	111.0	83.1	70.6	67.7	64.5	89.1	61.9	43.6	90.7	61.6	120.7	93.4
10:2 FTS	68.6	115.4	60.4	72.0	56.9	80.6	61.3	79.0	77.2	44.2	120.3	67.8	111.0	83.1	70.6	67.7	64.5	89.1	61.9	43.6	90.7	61.6	120.7	93.4

Table S5. Extractable internal standard (EIS) recoveries (%) of PFAS donor liver extracts for the human liver PFAS LC-MS/MS data. Values outside of the set EIS threshold (%) did not pass QC/QA and were not reported in the dataset.

Donor Number	PFBA	PFHxA	PFHpA	PFOA_TOF MS	PFNA	PFDA	PFUdA	PFDoA	PFTrDA	PFTeDA	5:3 FTCA	7:3 FTCA	PFBS	PFPeS	L-PFHxS_TOF MS	Br-PFHxS_TOF MS	PFHpS	L-PFOS_TOF MS	Br-PFOS_TOF MS	PFNS	PFDS	FOSA	L-N-MeFOSAA	Br-N-MeFOSAA	L-N-EtFOSAA	DONA	9ClPF3ONS	11ClPF3OUdS	3,6-OPFHpA	PFECHS	4:2 FTS	6:2 FTS	8:2 FTS	10:2 FTS
1	82.5	80.7	72.3	77.4	61.1	81.8	56.9	70.7	70.7	93.1	83.6	68.4	91.6	91.6	93.9	93.9	93.9	79.7	79.7	87.4	87.4	75.6	101.1	101.1	96.4	83.6	83.4	83.4	83.4	90.4	112.1	112.2	112.3	112.3
2	81.0	83.5	86.2	83.6	67.3	84.1	64.7	40.3	40.3	75.5	80.6	70.8	104.2	104.2	107.0	107.0	107.0	75.5	75.5	88.7	88.7	52.1	69.7	69.7	68.7	80.6	83.7	83.7	83.7	87.5	87.6	87.9	93.9	93.9
3	80.1	91.4	83.0	78.2	66.9	65.6	61.1	49.8	49.8	30.1	98.1	59.3	97.4	97.4	91.8	91.8	91.8	75.5	75.5	88.0	88.0	64.3	70.6	70.6	56.4	98.1	76.0	76.0	76.0	85.9	84.5	66.5	70.1	70.1
4	87.4	78.4	80.9	85.5	57.6	79.8	65.7	75.8	75.8	75.4	88.8	64.5	91.5	91.5	98.1	98.1	98.1	79.9	79.9	87.9	87.9	67.0	91.8	91.8	88.3	88.8	89.8	89.8	89.8	94.6	77.9	78.9	84.2	84.2
5	74.0	66.8	74.7	73.8	51.0	60.8	54.5	36.2	36.2	67.4	72.0	57.2	84.8	84.8	86.5	86.5	86.5	68.8	68.8	78.7	78.7	53.6	83.7	83.7	77.5	72.0	84.4	84.4	84.4	83.0	72.1	80.6	74.0	74.0
6	82.4	82.0	87.0	83.9	78.7	69.4	72.4	61.7	61.7	11.1	78.3	59.8	86.6	86.6	91.3	91.3	91.3	79.9	79.9	81.5	81.5	59.7	59.6	59.6	41.0	78.3	82.8	82.8	82.8	89.8	75.8	84.5	71.8	71.8
7	83.6	89.6	90.9	83.0	68.0	71.8	80.1	73.4	73.4	43.2	92.8	67.9	89.0	89.0	91.3	91.3	91.3	81.0	81.0	80.8	80.8	73.6	80.7	80.7	60.3	92.8	85.4	85.4	85.4	98.5	76.0	71.7	71.2	71.2
8	69.1	74.4	74.0	67.2	52.5	74.6	62.5	50.7	50.7	48.8	70.5	53.0	85.7	85.7	79.1	79.1	79.1	63.1	63.1	67.9	67.9	52.1	97.7	97.7	69.4	70.5	71.4	71.4	71.4	80.9	75.9	64.6	73.8	73.8
9	72.5	71.7	73.7	72.1	59.7	68.9	62.9	56.8	56.8	59.1	74.0	57.2	82.9	82.9	88.9	88.9	88.9	68.7	68.7	78.0	78.0	57.3	80.8	80.8	72.0	74.0	75.6	75.6	75.6	85.0	75.0	79.2	66.5	66.5
10	89.5	94.3	87.0	90.3	78.2	109.5	81.0	91.3	91.3	90.8	97.4	76.1	97.9	97.9	98.1	98.1	98.1	86.2	86.2	88.8	88.8	82.8	102.8	102.8	85.9	97.4	97.4	97.4	97.4	100.9	136.5	128.8	110.0	110.0
11	81.8	80.1	79.6	80.9	70.2	83.1	77.9	55.3	55.3	65.7	85.2	72.2	95.6	95.6	92.6	92.6	92.6	77.1	77.1	85.2	85.2	62.4	94.8	94.8	85.9	85.2	85.1	85.1	85.1	92.8	83.0	78.7	96.7	96.7
12	81.8	92.1	90.4	79.3	75.0	67.9	79.5	65.0	65.0	110.1	78.2	64.7	77.8	77.8	77.4	77.4	77.4	74.3	74.3	75.9	75.9	53.0	74.2	74.2	69.5	78.2	81.8	81.8	81.8	89.0	72.4	64.1	53.1	53.1
13	81.6	77.0	99.0	85.9	86.7	92.2	78.2	80.8	80.8	72.2	107.2	77.5	80.1	80.1	99.0	99.0	99.0	83.9	83.9	92.5	92.5	71.1	82.0	82.0	73.2	107.2	92.9	92.9	92.9	95.0	86.0	84.9	78.1	78.1
14	84.7	78.7	87.8	91.7	82.2	86.6	84.3	95.3	95.3	80.3	91.2	66.3	75.7	75.7	82.8	82.8	82.8	84.1	84.1	81.2	81.2	64.5	67.3	67.3	65.0	91.2	89.1	89.1	89.1	95.5	80.5	75.4	62.9	62.9
15	70.3	71.8	79.0	72.7	65.8	87.8	85.9	53.2	53.2	62.1	91.1	82.8	74.2	74.2	86.4	86.4	86.4	74.6	74.6	80.5	80.5	67.4	112.9	112.9	102.0	91.1	81.1	81.1	81.1	88.2	119.3	160.7	126.8	126.8
16	67.3	69.6	74.4	73.8	51.4	64.0	67.4	55.6	55.6	47.6	67.4	48.5	73.0	73.0	89.3	89.3	89.3	69.7	69.7	77.9	77.9	68.4	74.3	74.3	61.2	67.4	71.8	71.8	71.8	79.9	76.6	79.9	74.1	74.1
17	53.0	53.5	59.8	55.0	45.2	67.6	53.3	53.4	53.4	21.1	60.9	45.8	54.6	54.6	60.8	60.8	60.8	52.5	52.5	54.0	54.0	38.4	47.2	47.2	38.3	60.9	64.4	64.4	64.4	69.0	55.7	49.5	42.9	42.9
18	73.6	73.9	74.4	72.2	26.9	71.3	58.4	36.3	36.3	34.1	68.2	26.8	105.6	105.6	134.2	134.2	134.2	62.8	62.8	74.4	74.4	85.3	117.9	117.9	107.4	68.2	76.8	76.8	76.8	130.5	143.0	150.0	138.9	138.9
19	67.8	70.8	74.6	72.5	49.6	61.5	37.1	41.7	41.7	46.2	81.4	63.0	78.8	78.8	93.0	93.0	93.0	73.3	73.3	81.9	81.9	56.1	71.7	71.7	58.1	81.4	70.6	70.6	70.6	80.0	87.8	80.9	66.8	66.8
20	75.5	78.1	85.9	83.1	67.1	79.2	59.3	44.3	44.3	68.1	98.2	73.1	80.3	80.3	90.2	90.2	90.2	78.8	78.8	81.4	81.4	61.3	98.7	98.7	79.3	98.2	88.9	88.9	88.9	96.9	102.7	119.5	78.7	78.7
21	77.1	75.8	86.6	78.5	69.6	78.3	61.1	70.8	70.8	85.4	80.8	68.2	73.3	73.3	87.2	87.2	87.2	74.5	74.5	83.3	83.3	71.4	73.4	73.4	60.7	80.8	75.0	75.0	75.0	79.4	275.4	93.9	72.8	72.8
22	89.8	83.6	86.2	86.7	90.6	106.5	95.8	89.0	89.0	158.9	81.2	81.4	88.7	88.7	91.6	91.6	91.6	86.2	86.2	86.8	86.8	82.3	86.0	86.0	80.4	81.2	77.8	77.8	77.8	86.6	113.8	102.3	123.2	123.2
23	76.7	76.6	89.7	81.0	53.5	61.1	57.8	43.8	43.8	60.6	83.8	54.0	78.9	78.9	93.5	93.5	93.5	80.6	80.6	89.0	89.0	69.3	73.1	73.1	58.0	83.8	73.7	73.7	73.7	84.1	82.2	78.8	62.7	62.7
24	93.7	79.6	75.9	91.9	69.1	82.9	77.0	76.0	76.0	36.3	104.9	79.9	91.9	91.9	92.9	92.9	92.9	86.9	86.9	83.0	83.0	73.4	89.3	89.3	73.6	104.9	99.2	99.2	99.2	102.8	100.3	104.5	82.4	82.4
25	87.7	87.0	85.8	88.1	78.6	87.2	53.4	60.6	60.6	79.0	91.5	84.6	76.8	76.8	86.9	86.9	86.9	90.8	90.8	79.6	79.6	57.4	88.2	88.2	74.5	91.5	91.7	91.7	91.7	102.3	87.9	90.1	73.1	73.1
26	89.8	82.1	77.9	91.7	98.7	92.3	76.2	58.1	58.1	127.6	79.9	94.4	85.3	85.3	91.9	91.9	91.9	90.0	90.0	85.6	85.6	70.2	94.3	94.3	92.3	79.9	86.1	86.1	86.1	93.5	112.4	100.6	117.7	117.7
27	73.9	80.3	82.0	78.6	64.9	68.4	57.9	44.6	44.6	59.6	70.2	58.7	71.6	71.6	81.4	81.4	81.4	76.4	76.4	71.7	71.7	51.8	85.0	85.0	72.8	70.2	82.0	82.0	82.0	89.1	88.1	84.9	66.7	66.7
28	88.5	82.1	91.2	90.1	88.6	100.8	68.5	43.4	43.4	38.5	95.9	96.1	83.9	83.9	90.4	90.4	90.4	86.4	86.4	84.5	84.5	64.6	79.9	79.9	75.7	95.9	80.6	80.6	80.6	86.3	104.9	92.5	113.0	113.0
29	84.0	85.0	85.1	87.5	80.7	78.5	55.7	47.2	47.2	52.0	103.5	70.8	86.6	86.6	100.2	100.2	100.2	88.8	88.8	89.6	89.6	61.9	70.2	70.2	59.3	103.5	85.2	85.2	85.2	92.8	126.8	92.6	78.6	78.6
30	88.8	84.8	90.1	91.8	85.9	76.1	58.2	59.8	59.8	68.5	83.9	73.8	84.7	84.7	100.7	100.7	100.7	92.3	92.3	93.0	93.0	62.6	80.4	80.4	67.5	83.9	96.9	96.9	96.9	99.8	98.8	91.1	83.2	83.2
31	78.2	73.8	71.2	77.1	70.6	71.6	74.2	77.9	77.9	60.5	77.9	67.4	80.6	80.6	82.6	82.6	82.6	72.4	72.4	80.0	80.0	63.5	74.4	74.4	64.9	77.9	72.0	72.0	72.0	72.4	89.8	106.6	68.4	68.4
32	85.3	88.2	79.4	86.5	60.7	96.4	80.9	87.6	87.6	112.4	91.5	74.3	105.0	105.0	104.2	104.2	104.2	83.1	83.1	92.1	92.1	79.5	107.4	107.4	100.7	91.5	88.5	88.5	88.5	99.3	120.3	123.5	118.2	118.2
33	77.5	69.2	70.3	76.0	56.6	73.6	69.8	65.3	65.3	48.2	75.6	65.3	82.6	82.6	85.7	85.7	85.7	72.3	72.3	78.1	78.1	67.9	97.0	97.0	85.4	75.6	80.8	80.8	80.8	83.6	93.9	97.8	73.1	73.1
34	79.5	78.7	77.0	79.6	63.7	83.7	87.5	80.5	80.5	73.5	84.2	61.8	88.2	88.2	90.3	90.3	90.3	75.5	75.5	80.1	80.1	74.3	87.8	87.8	92.6	84.2	78.3	78.3	78.3	83.9	116.8	125.4	99.0	99.0
35	88.7	89.0	79.7	88.2	76.3	107.9	89.9	71.3	71.3	56.3	86.7	61.6	102.9	102.9	104.4	104.4	104.4	81.2	81.2	91.1	91.1	75.0	113.2	113.2	106.5	86.7	93.0	93.0	93.0	101.4	115.1	127.8	99.7	99.7
36	73.5	75.0	70.2	78.3	62.7	77.4	76.6	65.2	65.2	134.1	80.5	72.6	79.6	79.6	81.1	81.1	81.1	74.0	74.0	80.7	80.7	70.2	84.5	84.5	81.9	80.5	80.5	80.5	80.5	81.6	73.5	72.7	77.7	77.7
37	73.5	81.0	75.0	75.9	65.3	65.8	62.9	46.4	46.4	61.2	75.3	61.7	82.7	82.7	81.7	81.7	81.7	73.7	73.7	70.2	70.2	55.1	72.5	72.5	63.2	75.3	77.9	77.9	77.9	82.9	78.8	59.9	58.1	58.1
38	80.1	84.1	87.6	84.1	73.8	80.4	79.3	68.9	68.9	91.2	91.0	86.4	80.2	80.2	79.9	79.9	79.9	82.0	82.0	84.3	84.3	61.8	76.7	76.7	76.9	91.0	85.8	85.8	85.8	88.9	80.9	69.4	70.3	70.3
39	83.8	93.9	105.6	95.2	92.1	119.6	96.1	63.1	63.1	69.7	99.0	83.2	91.8	91.8	107.8	107.8	107.8	92.9	92.9	109.7	109.7	64.8	87.8	87.8	78.7	99.0	103.2	103.2	103.2	109.4	134.0	101.8	84.2	84.2
40	86.3	100.6	102.9	96.4	87.0	86.9	79.1	65.2	65.2	109.4	98.1	75.3	101.7	101.7	108.9	108.9	108.9	92.9	92.9	112.1	112.1	83.1	80.1	80.1	68.8	98.1	90.8	90.8	90.8	91.2	111.6	86.5	86.7	86.7
41	86.7	97.1	86.2	90.5	72.7	104.5	68.8	92.3	92.3	133.1	113.1	83.2	109.1	109.1	104.6	104.6	104.6	85.3	85.3	95.4	95.4	90.3	87.3	87.3	81.3	113.1	84.4	84.4	84.4	95.6	100.0	66.1	63.0	63.0
42	89.8	102.6	109.2	90.0	110.8	98.6	73.4	56.2	56.2	58.6	87.2	83.2	83.6	83.6	101.1	101.1	101.1	89.8	89.8	90.4	90.4	53.1	78.5	78.5	66.4	87.2	96.0	96.0	96.0	102.9	118.5	100.4	66.6	66.6
43	84.2	90.4	90.2	91.4	76.8	62.5	61.3	54.5	54.5	70.5	95.4	95.3	85.2	85.2	89.2	89.2	89.2	90.1	90.1	85.8	85.8	53.3	67.8	67.8	60.6	95.4	99.6	99.6	99.6	101.7	90.3	74.1	74.5	74.5
44	80.0	59.2	95.6	79.9	67.8	78.0	67.5	40.1	40.1	97.4	84.5	75.9	75.0	75.0	86.3	86.3	86.3	75.2	75.2	80.6	80.6	50.5	74.0	74.0	64.9	84.5	82.6	82.6	82.6	83.8	205.3	64.2	59.1	59.1
45	63.5	60.9	62.1	66.1	47.7	59.5	58.3	48.2	48.2	57.6	76.5	58.3	67.5	67.5	81.8	81.8	81.8	61.8	61.8	72.8	72.8	63.6	75.1	75.1	59.9	76.5	70.0	70.0	70.0	75.3	83.0	67.7	66.7	66.7
46	93.5	91.0	84.3	98.4	77.1	97.6	87.8	78.7	78.7	204.3	87.9	88.1	98.9	98.9	103.2	103.2	103.2	94.1	94.1	84.1	84.1	75.2	99.6	99.6	84.7	87.9	88.8	88.8	88.8	105.4	133.0	124.8	105.4	105.4
47	82.1	69.8	78.3	85.6	71.8	82.1	76.6	66.9	66.9	63.9	90.5	89.4	75.8	75.8	92.6	92.6	92.6	80.8	80.8	89.5	89.5	59.7	87.5	87.5	69.5	90.5	102.2	102.2	102.2	103.1	80.1	71.4	73.9	73.9
48	61.7	60.4	61.5	60.6	53.9	59.1	39.6	68.9	68.9	64.9	56.1	48.6	63.3	63.3	76.9	76.9	76.9	61.3	61.3	71.8	71.8	60.8	71.1	71.1	61.2	56.1	71.9	71.9	71.9	76.7	102.4	66.1	61.4	61.4
49	70.9	71.6	81.3	74.0	49.9	61.8	59.4	54.9	54.9	60.8	84.4	47.9	70.6	70.6	83.5	83.5	83.5	70.4	70.4	68.0	68.0	64.8	78.7	78.7	68.3	84.4	79.6	79.6	79.6	89.2	71.5	74.6	61.2	61.2
50	89.7	78.4	78.7	89.4	70.4	92.3	94.8	73.3	73.3	83.0	76.9	56.3	83.9	83.9	102.8	102.8	102.8	86.3	86.3	90.5	90.5	70.3	99.0	99.0	84.9	76.9	92.4	92.4	92.4	109.0	73.7	87.6	79.0	79.0
51	96.2	92.9	115.5	95.5	83.6	120.5	84.8	69.0	69.0	90.4	85.5	57.5	94.7	94.7	111.3	111.3	111.3	91.3	91.3	99.0	99.0	75.6	96.6	96.6	87.6	85.5	96.2	96.2	96.2	105.7	116.2	110.4	96.6	96.6
52	59.0	52.7	56.7	60.3	46.7	45.3	61.3	39.5	39.5	38.6	70.5	60.8	57.3	57.3	69.1	69.1	69.1	60.5	60.5	67.6	67.6	44.6	66.2	66.2	57.2	70.5	64.7	64.7	64.7	66.6	61.6	57.8	52.7	52.7
53	90.8	88.5	97.9	93.3	83.9	120.9	110.7	92.9	92.9	166.7	88.3	75.2	84.7	84.7	93.8	93.8	93.8	84.0	84.0	75.9	75.9	77.4	88.4	88.4	78.3	88.3	78.9	78.9	78.9	98.7	105.6	98.8	88.8	88.8
54	88.7	84.5	92.8	98.5	60.7	84.4	76.5	64.8	64.8	40.1	95.8	72.4	95.8	95.8	108.7	108.7	108.7	99.5	99.5	99.4	99.4	69.4	85.7	85.7	66.5	95.8	100.7	100.7	100.7	103.7	118.6	103.2	78.5	78.5
55	65.1	73.9	88.5	70.2	65.6	70.6	67.5	50.6	50.6	47.4	66.4	47.4	71.6	71.6	81.0	81.0	81.0	67.0	67.0	69.6	69.6	53.1	75.0	75.0	62.0	66.4	69.9	69.9	69.9	85.3	79.9	79.8	65.1	65.1
56	72.7	74.2	83.1	79.2	70.6	77.3	47.7	41.7	41.7	34.9	80.7	62.6	71.5	71.5	80.7	80.7	80.7	75.8	75.8	75.5	75.5	41.8	73.1	73.1	64.3	80.7	85.2	85.2	85.2	85.0	83.9	74.9	58.2	58.2
57	68.6	71.0	71.3	66.9	64.5	70.4	74.9	43.8	43.8	60.6	70.9	58.1	62.9	62.9	73.9	73.9	73.9	68.2	68.2	68.7	68.7	55.3	76.2	76.2	60.4	70.9	71.7	71.7	71.7	74.4	67.5	80.0	62.7	62.7
58	79.6	83.4	94.0	87.5	62.4	84.0	74.0	40.4	40.4	36.2	95.1	83.0	76.9	76.9	94.2	94.2	94.2	84.8	84.8	87.3	87.3	62.4	90.5	90.5	85.5	95.1	91.7	91.7	91.7	97.1	89.5	83.9	77.0	77.0
59	91.2	82.2	83.7	92.2	87.4	94.7	100.7	67.0	67.0	123.5	91.2	89.3	87.0	87.0	96.3	96.3	96.3	88.9	88.9	83.5	83.5	68.2	91.6	91.6	88.3	91.2	92.0	92.0	92.0	99.4	102.5	98.3	89.3	89.3
60	74.5	67.1	72.8	80.4	53.2	74.6	65.0	49.3	49.3	38.2	93.3	55.4	76.1	76.1	92.5	92.5	92.5	77.7	77.7	83.7	83.7	56.1	69.1	69.1	53.8	93.3	82.9	82.9	82.9	89.8	84.1	84.7	73.5	73.5
61	88.0	83.2	69.6	91.0	88.6	105.8	98.5	79.9	79.9	162.7	80.8	99.3	80.8	80.8	88.7	88.7	88.7	89.8	89.8	79.1	79.1	80.1	91.8	91.8	80.3	80.8	85.5	85.5	85.5	91.3	116.4	107.7	102.8	102.8
62	86.3	83.6	80.8	88.6	83.0	95.9	75.4	74.0	74.0	165.4	75.6	77.2	91.7	91.7	93.5	93.5	93.5	84.7	84.7	82.8	82.8	66.9	87.9	87.9	80.4	75.6	77.9	77.9	77.9	96.4	132.9	108.0	96.7	96.7
63	83.8	87.9	93.5	88.9	58.2	78.8	48.1	44.6	44.6	62.9	87.4	66.1	101.3	101.3	115.3	115.3	115.3	78.4	78.4	83.4	83.4	58.7	93.5	93.5	80.4	87.4	99.8	99.8	99.8	115.1	118.4	96.7	79.2	79.2
64	91.8	87.9	75.7	91.8	94.2	100.7	85.3	71.5	71.5	135.1	81.3	91.5	83.9	83.9	89.3	89.3	89.3	87.1	87.1	84.3	84.3	72.1	75.4	75.4	69.9	81.3	80.8	80.8	80.8	86.8	111.8	100.0	120.4	120.4
65	84.6	83.7	87.1	88.9	79.2	107.2	95.3	67.5	67.5	105.1	87.6	80.1	102.0	102.0	103.0	103.0	103.0	84.2	84.2	96.5	96.5	77.2	97.9	97.9	88.8	87.6	88.9	88.9	88.9	98.0	130.9	112.9	126.3	126.3
66	89.7	92.0	98.6	92.1	74.1	102.4	80.6	36.7	36.7	57.5	102.4	85.7	77.8	77.8	96.7	96.7	96.7	87.7	87.7	86.3	86.3	53.3	70.7	70.7	50.9	102.4	91.6	91.6	91.6	101.5	89.5	82.8	71.2	71.2
67	84.1	89.6	85.9	79.8	62.7	88.3	80.0	75.2	75.2	51.5	89.9	68.2	102.8	102.8	99.1	99.1	99.1	75.7	75.7	91.3	91.3	78.4	80.4	80.4	60.3	89.9	79.4	79.4	79.4	85.3	109.0	106.8	98.8	98.8
68	74.9	77.6	72.8	72.0	72.2	73.6	75.0	78.2	78.2	90.7	84.9	67.1	74.2	74.2	77.0	77.0	77.0	72.0	72.0	72.5	72.5	48.9	67.3	67.3	64.2	84.9	72.3	72.3	72.3	69.0	86.0	79.9	56.8	56.8
69	83.1	72.0	71.8	78.0	68.2	84.4	68.8	61.9	61.9	19.7	73.8	71.1	90.4	90.4	90.4	90.4	90.4	75.5	75.5	81.3	81.3	60.4	133.1	133.1	125.4	73.8	78.6	78.6	78.6	87.8	89.4	85.5	133.5	133.5
70	80.4	67.0	65.3	73.1	73.6	73.2	62.6	43.2	43.2	13.0	68.4	72.5	83.8	83.8	86.4	86.4	86.4	67.8	67.8	79.4	79.4	50.9	103.6	103.6	90.9	68.4	77.4	77.4	77.4	82.4	83.4	76.8	101.7	101.7
71	80.4	61.1	54.4	76.1	62.1	55.4	43.4	27.0	27.0	30.4	70.7	68.5	93.5	93.5	84.7	84.7	84.7	72.8	72.8	75.1	75.1	16.8	55.9	55.9	52.2	70.7	89.1	89.1	89.1	105.2	158.5	256.3	157.9	157.9
72	70.7	86.5	85.3	79.4	67.5	80.3	68.4	71.7	71.7	63.0	81.3	64.1	85.1	85.1	88.0	88.0	88.0	77.6	77.6	87.4	87.4	67.5	62.5	62.5	51.7	81.3	87.0	87.0	87.0	85.1	90.8	65.3	50.7	50.7
73	77.9	74.4	72.6	77.8	69.4	92.9	76.4	85.5	85.5	108.8	75.1	59.8	96.1	96.1	96.5	96.5	96.5	75.4	75.4	83.4	83.4	72.8	82.7	82.7	81.0	75.1	85.4	85.4	85.4	84.0	99.6	163.9	80.7	80.7
74	63.3	59.2	53.3	61.0	47.8	48.5	51.5	54.4	54.4	92.4	62.1	51.3	67.3	67.3	67.5	67.5	67.5	57.9	57.9	62.9	62.9	41.3	64.9	64.9	58.1	62.1	67.2	67.2	67.2	69.4	78.3	82.0	48.9	48.9
75	73.9	75.4	65.9	70.1	68.7	67.6	64.0	75.7	75.7	37.5	76.2	64.9	71.8	71.8	75.7	75.7	75.7	63.4	63.4	76.7	76.7	50.0	66.6	66.6	60.2	76.2	75.4	75.4	75.4	75.8	77.2	76.9	66.2	66.2
76	85.7	90.3	104.5	90.5	74.4	92.3	80.9	54.1	54.1	71.4	103.0	91.1	84.4	84.4	88.2	88.2	88.2	88.3	88.3	90.5	90.5	64.6	63.3	63.3	57.4	103.0	87.5	87.5	87.5	82.7	82.3	73.7	60.9	60.9
77	90.4	125.0	116.3	100.8	98.2	91.5	89.8	67.5	67.5	96.8	110.2	76.1	103.7	103.7	103.7	103.7	103.7	100.0	100.0	106.2	106.2	83.7	71.7	71.7	66.8	110.2	106.5	106.5	106.5	103.5	110.5	75.2	65.3	65.3
78	71.0	64.6	59.0	70.2	55.0	68.7	68.8	73.4	73.4	43.9	72.0	64.6	73.1	73.1	76.3	76.3	76.3	66.5	66.5	69.2	69.2	53.5	137.1	137.1	117.6	72.0	73.5	73.5	73.5	73.2	81.9	85.2	78.8	78.8
79	83.4	99.3	97.6	96.4	80.4	100.3	103.2	82.8	82.8	102.1	105.2	78.5	101.6	101.6	108.3	108.3	108.3	93.6	93.6	109.4	109.4	93.0	73.7	73.7	62.8	105.2	100.9	100.9	100.9	99.4	104.5	79.1	68.1	68.1
80	90.8	104.2	105.8	99.1	81.4	97.7	89.1	80.8	80.8	93.3	86.4	79.1	77.8	77.8	85.8	85.8	85.8	96.7	96.7	85.3	85.3	59.1	69.2	69.2	65.4	86.4	101.2	101.2	101.2	93.2	91.3	67.9	57.5	57.5
81	81.8	98.1	88.5	91.6	79.8	78.2	96.3	76.5	76.5	62.5	97.0	89.3	81.0	81.0	87.3	87.3	87.3	88.0	88.0	88.6	88.6	66.7	60.2	60.2	57.0	97.0	83.7	83.7	83.7	79.8	81.7	58.1	43.5	43.5
82	76.4	73.9	74.5	81.2	68.3	64.6	66.7	54.5	54.5	68.2	93.8	78.1	82.8	82.8	89.2	89.2	89.2	77.7	77.7	88.5	88.5	63.8	76.9	76.9	81.7	93.8	89.1	89.1	89.1	94.6	97.9	88.8	84.2	84.2
83	65.6	67.1	59.5	63.8	64.6	59.1	69.6	61.7	61.7	71.8	78.5	64.3	63.5	63.5	64.2	64.2	64.2	61.3	61.3	62.1	62.1	42.6	75.8	75.8	62.4	78.5	77.0	77.0	77.0	75.7	72.3	76.8	48.0	48.0
84	85.7	96.6	103.9	95.5	89.4	85.1	91.4	82.4	82.4	87.2	105.5	95.6	93.7	93.7	93.5	93.5	93.5	91.4	91.4	98.7	98.7	67.2	75.3	75.3	76.8	105.5	96.8	96.8	96.8	88.4	92.6	102.4	77.0	77.0
85	78.1	84.5	90.7	81.4	71.6	78.4	74.1	44.6	44.6	33.3	76.1	63.8	79.7	79.7	94.7	94.7	94.7	84.0	84.0	77.1	77.1	52.6	76.5	76.5	67.4	76.1	91.1	91.1	91.1	93.2	85.6	93.2	70.0	70.0
86	74.6	80.8	88.6	78.6	59.3	77.8	73.8	58.8	58.8	53.2	80.5	63.6	84.9	84.9	86.6	86.6	86.6	73.9	73.9	89.3	89.3	57.4	59.0	59.0	52.1	80.5	82.9	82.9	82.9	83.6	77.2	58.3	44.9	44.9
87	85.5	70.2	78.6	90.2	70.3	75.9	85.7	60.7	60.7	61.2	90.1	78.3	71.8	71.8	86.0	86.0	86.0	87.4	87.4	82.5	82.5	63.3	71.0	71.0	64.3	90.1	85.0	85.0	85.0	87.3	76.4	77.0	66.0	66.0
88	85.5	91.7	90.4	93.6	91.5	80.0	92.6	60.4	60.4	40.5	100.5	100.0	82.2	82.2	91.6	91.6	91.6	79.1	79.1	93.3	93.3	56.3	84.2	84.2	70.2	100.5	103.8	103.8	103.8	93.8	107.7	128.6	122.1	122.1
89	85.8	88.3	83.6	81.6	69.4	90.6	87.3	87.7	87.7	83.7	85.7	79.8	95.3	95.3	94.0	94.0	94.0	80.5	80.5	87.0	87.0	61.2	91.3	91.3	81.5	85.7	89.4	89.4	89.4	86.5	111.9	107.8	72.1	72.1
90	93.3	86.4	96.7	95.9	74.7	71.1	64.1	77.1	77.1	75.1	94.2	91.4	88.8	88.8	95.1	95.1	95.1	94.1	94.1	94.4	94.4	64.9	77.7	77.7	79.2	94.2	105.5	105.5	105.5	103.6	91.3	78.9	66.4	66.4
91	75.6	64.8	64.1	71.5	61.6	77.8	71.7	46.9	46.9	48.9	67.5	63.3	79.5	79.5	83.1	83.1	83.1	67.6	67.6	73.7	73.7	49.6	112.0	112.0	115.4	67.5	75.5	75.5	75.5	82.7	81.3	79.2	98.6	98.6
92	71.5	64.2	64.5	68.3	61.7	70.0	69.9	76.9	76.9	101.6	67.8	65.5	78.1	78.1	76.9	76.9	76.9	63.2	63.2	76.3	76.3	53.2	68.8	68.8	63.8	67.8	74.3	74.3	74.3	71.6	82.7	84.9	59.8	59.8
93	76.9	90.7	83.2	85.5	70.7	70.0	55.9	60.7	60.7	80.8	96.7	88.3	99.9	99.9	104.9	104.9	104.9	84.0	84.0	102.4	102.4	74.8	51.3	51.3	45.6	96.7	95.1	95.1	95.1	89.5	105.1	73.9	60.2	60.2
94	87.9	94.1	103.6	99.9	87.4	104.1	95.3	75.0	75.0	58.7	91.1	87.4	88.8	88.8	92.6	92.6	92.6	93.3	93.3	93.4	93.4	62.4	79.8	79.8	74.3	91.1	95.2	95.2	95.2	89.5	98.1	133.3	81.1	81.1
95	74.4	99.8	105.5	86.4	79.1	58.4	65.9	49.3	49.3	77.9	83.5	72.4	95.7	95.7	94.6	94.6	94.6	82.7	82.7	95.3	95.3	52.2	56.2	56.2	50.4	83.5	96.4	96.4	96.4	89.0	84.4	76.9	46.8	46.8
96	74.7	81.4	87.0	80.5	68.1	72.0	66.9	74.0	74.0	86.3	73.5	62.5	89.3	89.3	89.2	89.2	89.2	78.7	78.7	85.7	85.7	71.1	55.7	55.7	51.9	73.5	85.5	85.5	85.5	79.6	84.7	99.7	51.4	51.4
97	89.1	73.5	70.7	85.9	79.2	69.4	77.0	70.2	70.2	69.7	92.4	81.3	99.9	99.9	99.9	99.9	99.9	81.7	81.7	100.1	100.1	78.1	97.3	97.3	86.7	92.4	94.9	94.9	94.9	89.8	110.5	166.2	91.0	91.0
98	74.2	76.5	74.6	78.5	73.5	58.4	62.8	52.3	52.3	55.8	78.4	70.9	81.8	81.8	82.7	82.7	82.7	74.1	74.1	79.9	79.9	60.4	67.1	67.1	84.6	78.4	90.9	90.9	90.9	87.5	87.8	88.4	84.2	84.2
99	87.9	100.6	102.1	96.0	76.2	100.9	88.4	85.9	85.9	147.2	104.0	93.2	98.3	98.3	101.0	101.0	101.0	95.3	95.3	109.0	109.0	83.1	70.2	70.2	61.8	104.0	102.6	102.6	102.6	98.3	97.1	66.1	70.8	70.8
100	66.1	59.6	58.7	63.9	67.9	68.1	79.8	73.7	73.7	50.2	58.5	73.1	67.8	67.8	68.2	68.2	68.2	60.0	60.0	66.5	66.5	48.0	79.0	79.0	80.2	58.5	69.7	69.7	69.7	65.6	62.5	50.4	58.5	58.5
101	79.1	88.0	86.1	87.2	73.7	91.3	68.8	64.4	64.4	94.4	99.2	88.2	79.6	79.6	82.2	82.2	82.2	88.1	88.1	87.3	87.3	64.2	61.7	61.7	53.1	99.2	86.4	86.4	86.4	87.5	76.7	57.0	50.4	50.4
102	76.2	101.4	102.3	88.2	76.6	76.5	72.7	57.9	57.9	56.1	96.7	96.6	108.8	108.8	115.5	115.5	115.5	85.7	85.7	103.9	103.9	72.4	55.9	55.9	49.8	96.7	84.1	84.1	84.1	85.7	116.6	73.9	65.7	65.7
103	76.4	82.7	94.2	77.3	82.7	86.5	83.3	61.1	61.1	49.6	68.1	56.7	79.1	79.1	89.2	89.2	89.2	75.3	75.3	82.3	82.3	55.1	62.1	62.1	57.6	68.1	81.2	81.2	81.2	85.9	78.9	82.7	70.2	70.2
104	89.2	88.0	82.2	87.3	78.4	95.6	77.9	83.4	83.4	103.2	84.0	86.6	88.5	88.5	89.9	89.9	89.9	83.5	83.5	87.2	87.2	64.5	95.6	95.6	85.0	84.0	95.7	95.7	95.7	89.5	96.1	97.7	73.5	73.5
105	90.1	101.7	117.3	101.0	87.5	104.4	94.0	78.1	78.1	125.0	103.8	85.0	95.9	95.9	101.7	101.7	101.7	97.2	97.2	97.0	97.0	77.2	65.8	65.8	57.5	103.8	99.2	99.2	99.2	98.3	103.7	69.4	54.4	54.4

Donor Number	PFBA	PFHxA	PFHpA	PFOA_TOF MS	PFNA	PFDA	PFUdA	PFDoA	PFTrDA	PFTeDA	5:3 FTCA	7:3 FTCA	PFBS	PFPeS	L-PFHxS_TOF MS	Br-PFHxS_TOF MS	PFHpS	L-PFOS_TOF MS	Br-PFOS_TOF MS	PFNS	PFDS	FOSA	L-N-MeFOSAA	Br-N-MeFOSAA	L-N-EtFOSAA	DONA	9ClPF3ONS	11ClPF3OUdS	3,6-OPFHpA	PFECHS	4:2 FTS	6:2 FTS	8:2 FTS	10:2 FTS
105	90.1	101.7	117.3	101.0	87.5	104.4	94.0	78.1	78.1	125.0	103.8	85.0	95.9	95.9	101.7	101.7	101.7	97.2	97.2	97.0	97.0	77.2	65.8	65.8	57.5	103.8	99.2	99.2	99.2	98.3	103.7	69.4	54.4	54.4
106	77.2	93.8	85.6	83.0	65.3	83.5	75.6	72.6	72.6	87.9	95.4	69.0	84.4	84.4	83.8	83.8	83.8	77.6	77.6	87.2	87.2	73.6	60.5	60.5	49.8	95.4	87.5	87.5	87.5	88.1	88.2	59.7	51.6	51.6
107	40.8	35.6	35.8	36.6	42.6	38.4	34.5	25.7	25.7	0.3	36.4	38.1	41.0	41.0	42.4	42.4	42.4	33.7	33.7	37.1	37.1	28.2	23.7	23.7	15.5	36.4	39.0	39.0	39.0	41.3	46.8	36.3	38.2	38.2
108	79.4	79.0	78.9	83.1	68.7	104.0	61.3	62.3	62.3	69.4	85.1	62.8	80.4	80.4	82.7	82.7	82.7	79.7	79.7	79.9	79.9	66.2	93.3	93.3	76.7	85.1	82.9	82.9	82.9	85.8	84.5	77.9	82.2	82.2
109	77.2	63.3	70.4	82.3	54.9	92.3	75.6	47.6	47.6	54.2	72.4	75.8	66.6	66.6	77.3	77.3	77.3	78.9	78.9	74.7	74.7	50.4	65.5	65.5	60.8	72.4	80.0	80.0	80.0	82.6	66.1	115.2	56.6	56.6
110	81.8	72.7	72.9	77.6	87.2	100.9	85.6	83.5	83.5	109.8	78.3	87.8	82.6	82.6	84.6	84.6	84.6	73.9	73.9	83.3	83.3	54.3	109.4	109.4	106.0	78.3	87.5	87.5	87.5	83.8	89.4	74.3	93.0	93.0
111	68.7	59.2	72.0	73.5	64.7	72.7	74.5	64.8	64.8	46.7	68.7	63.2	58.7	58.7	68.7	68.7	68.7	70.7	70.7	67.5	67.5	46.8	60.3	60.3	57.8	68.7	73.3	73.3	73.3	69.9	61.2	67.4	54.5	54.5
112	74.7	65.2	61.7	69.7	61.8	86.4	108.5	97.2	97.2	16.4	65.6	68.1	79.2	79.2	80.2	80.2	80.2	63.3	63.3	77.1	77.1	67.7	67.9	67.9	51.0	65.6	71.0	71.0	71.0	72.9	79.2	65.7	77.6	77.6
113	74.3	67.9	63.6	67.9	75.4	88.0	87.2	86.7	86.7	48.6	60.6	71.1	70.0	70.0	69.1	69.1	69.1	63.7	63.7	71.9	71.9	51.8	73.8	73.8	69.3	60.6	66.3	66.3	66.3	66.3	65.1	61.4	54.8	54.8
114	27.2	21.9	22.5	22.2	24.1	25.1	22.4	18.8	18.8	0.2	20.2	22.2	24.6	24.6	24.4	24.4	24.4	19.7	19.7	24.5	24.5	18.6	10.7	10.7	6.8	20.2	23.3	23.3	23.3	26.2	26.3	22.2	23.2	23.2
115	79.3	90.8	87.5	89.8	58.8	75.2	65.2	71.3	71.3	72.9	85.6	74.2	88.8	88.8	93.3	93.3	93.3	84.5	84.5	95.6	95.6	67.2	64.3	64.3	59.9	85.6	100.3	100.3	100.3	97.6	97.1	69.6	59.0	59.0
116	79.8	92.9	85.9	86.7	73.8	81.5	61.9	48.7	48.7	42.0	98.1	82.7	87.4	87.4	82.4	82.4	82.4	86.4	86.4	83.1	83.1	50.7	54.0	54.0	49.8	98.1	97.6	97.6	97.6	93.7	82.5	58.2	45.9	45.9
117	77.0	71.1	67.5	70.8	57.7	87.4	86.9	89.9	89.9	60.5	67.5	59.7	94.1	94.1	92.0	92.0	92.0	65.1	65.1	77.4	77.4	56.4	126.4	126.4	123.3	67.5	76.7	76.7	76.7	92.6	97.0	85.1	98.4	98.4
118	34.8	28.1	27.7	29.0	30.1	29.8	31.8	27.7	27.7	0.4	28.1	30.9	33.8	33.8	33.0	33.0	33.0	25.4	25.4	30.2	30.2	22.0	15.3	15.3	8.9	28.1	30.8	30.8	30.8	30.2	37.4	25.9	27.5	27.5
119	85.3	82.0	84.0	93.0	74.1	103.7	72.3	62.5	62.5	33.2	91.8	88.1	83.5	83.5	99.9	99.9	99.9	90.8	90.8	92.2	92.2	63.7	83.3	83.3	65.9	91.8	94.4	94.4	94.4	96.3	94.3	87.7	80.1	80.1
120	78.8	69.3	67.2	75.5	79.7	80.9	95.7	107.5	107.5	102.4	66.3	81.6	74.8	74.8	75.3	75.3	75.3	72.0	72.0	77.9	77.9	57.7	96.8	96.8	105.9	66.3	75.9	75.9	75.9	73.2	70.1	67.1	71.8	71.8
121	28.3	22.9	21.9	24.0	23.5	21.2	19.6	14.0	14.0	0.2	22.5	25.1	29.1	29.1	29.0	29.0	29.0	20.5	20.5	24.1	24.1	16.9	7.8	7.8	2.9	22.5	24.1	24.1	24.1	28.0	33.3	22.9	21.8	21.8
122	48.6	40.3	41.9	42.7	47.8	49.0	41.8	36.7	36.7	0.1	42.5	46.1	47.3	47.3	47.8	47.8	47.8	38.6	38.6	45.1	45.1	31.0	14.5	14.5	5.7	42.5	43.9	43.9	43.9	45.0	51.2	45.5	47.9	47.9
123	76.1	91.1	87.5	78.1	82.2	77.6	76.3	73.8	73.8	54.8	104.2	91.4	83.6	83.6	85.5	85.5	85.5	81.0	81.0	89.1	89.1	60.5	53.5	53.5	57.3	104.2	88.6	88.6	88.6	81.7	84.7	56.1	46.4	46.4
124	87.2	86.7	85.4	92.1	59.9	79.3	53.4	51.6	51.6	112.4	110.3	80.7	107.7	107.7	106.9	106.9	106.9	86.7	86.7	114.0	114.0	78.8	55.8	55.8	53.4	110.3	90.4	90.4	90.4	93.6	108.0	149.7	61.6	61.6
125	79.9	84.9	82.1	85.5	67.6	62.8	53.9	51.3	51.3	89.7	92.2	79.2	92.3	92.3	96.2	96.2	96.2	82.9	82.9	88.3	88.3	68.1	46.1	46.1	45.5	92.2	74.2	74.2	74.2	75.1	104.0	65.1	51.3	51.3
126	71.9	78.7	83.7	80.3	74.3	71.0	65.0	59.4	59.4	79.6	87.0	67.7	73.1	73.1	78.9	78.9	78.9	73.6	73.6	77.7	77.7	66.8	63.9	63.9	70.1	87.0	88.3	88.3	88.3	88.0	89.6	85.1	59.6	59.6
127	70.6	69.3	78.3	76.6	75.2	63.6	60.1	40.1	40.1	49.4	80.2	68.1	73.6	73.6	86.2	86.2	86.2	72.8	72.8	85.2	85.2	50.9	68.3	68.3	55.5	80.2	81.0	81.0	81.0	82.6	80.0	98.5	74.8	74.8
128	86.7	91.1	102.0	94.3	85.0	95.6	85.4	81.1	81.1	38.6	97.7	87.4	82.9	82.9	95.2	95.2	95.2	94.8	94.8	84.7	84.7	59.4	80.0	80.0	64.3	97.7	91.4	91.4	91.4	93.8	92.3	79.9	71.0	71.0
129	84.5	79.2	83.9	87.6	80.0	70.3	57.6	45.2	45.2	33.3	90.2	85.5	91.4	91.4	94.9	94.9	94.9	88.6	88.6	97.4	97.4	56.6	68.9	68.9	68.3	90.2	92.3	92.3	92.3	85.2	99.8	88.4	78.8	78.8
130	69.4	64.2	62.4	64.3	70.0	71.9	75.6	73.2	73.2	26.0	64.1	69.0	72.5	72.5	72.0	72.0	72.0	62.2	62.2	68.3	68.3	52.3	71.5	71.5	62.9	64.1	66.0	66.0	66.0	64.7	58.6	48.2	48.9	48.9
131	80.7	77.9	69.9	76.5	53.8	69.7	75.5	91.9	91.9	106.5	85.2	57.4	91.0	91.0	93.0	93.0	93.0	72.4	72.4	82.6	82.6	65.7	95.2	95.2	88.6	85.2	85.8	85.8	85.8	96.7	111.3	113.1	75.9	75.9
132	76.1	90.2	81.5	84.6	76.1	63.4	49.2	32.1	32.1	20.4	90.0	83.2	90.5	90.5	90.1	90.1	90.1	78.3	78.3	85.7	85.7	46.5	46.9	46.9	41.3	90.0	85.0	85.0	85.0	89.1	92.4	51.3	40.9	40.9
133	77.6	87.4	80.2	80.9	77.1	56.8	71.9	63.9	63.9	50.9	91.6	76.4	80.9	80.9	81.1	81.1	81.1	79.6	79.6	76.3	76.3	65.5	50.3	50.3	45.4	91.6	85.2	85.2	85.2	80.9	85.7	48.7	46.4	46.4
134	70.3	70.7	78.5	76.1	61.7	68.0	73.1	65.6	65.6	45.8	71.4	57.7	74.1	74.1	86.7	86.7	86.7	73.4	73.4	81.3	81.3	49.7	73.0	73.0	61.0	71.4	84.7	84.7	84.7	84.3	88.1	99.3	71.8	71.8
135	79.5	62.9	73.6	81.6	82.7	59.6	59.7	62.0	62.0	46.0	83.3	88.3	80.6	80.6	95.7	95.7	95.7	83.7	83.7	88.0	88.0	58.8	90.6	90.6	61.6	83.3	81.0	81.0	81.0	82.7	98.8	108.3	86.7	86.7
136	68.2	69.6	65.0	61.7	62.9	56.6	54.7	50.3	50.3	61.3	64.6	64.1	63.1	63.1	63.4	63.4	63.4	60.2	60.2	65.6	65.6	38.9	62.4	62.4	55.3	64.6	63.1	63.1	63.1	63.1	75.7	70.4	47.2	47.2
137	78.0	73.8	83.6	76.1	69.0	77.2	68.9	56.7	56.7	35.5	80.4	67.6	69.0	69.0	81.9	81.9	81.9	77.3	77.3	81.8	81.8	52.6	76.9	76.9	62.5	80.4	80.6	80.6	80.6	83.3	81.2	79.9	63.5	63.5
138	70.6	62.3	58.8	66.7	64.4	77.4	91.4	79.6	79.6	21.6	61.7	66.3	71.0	71.0	70.6	70.6	70.6	61.4	61.4	69.4	69.4	59.7	80.9	80.9	66.0	61.7	72.1	72.1	72.1	74.4	72.0	65.9	82.1	82.1
139	81.7	92.9	103.2	91.4	75.9	101.1	125.6	76.8	76.8	146.4	88.9	66.2	87.3	87.3	104.2	104.2	104.2	89.0	89.0	88.3	88.3	80.9	93.8	93.8	85.0	88.9	95.6	95.6	95.6	106.1	102.1	97.6	95.0	95.0
140	82.2	69.9	70.1	74.4	78.5	81.2	95.0	91.7	91.7	29.2	68.2	78.8	82.2	82.2	81.0	81.0	81.0	71.7	71.7	78.7	78.7	55.0	88.7	88.7	84.9	68.2	80.0	80.0	80.0	76.9	70.3	79.5	56.1	56.1
141	67.8	59.4	58.2	62.6	65.5	62.9	56.6	58.8	58.8	29.8	58.2	68.5	73.5	73.5	70.9	70.9	70.9	59.5	59.5	64.6	64.6	49.1	73.8	73.8	73.9	58.2	67.3	67.3	67.3	66.7	61.0	53.1	56.3	56.3
142	46.6	37.2	35.2	39.7	40.7	44.2	40.4	38.7	38.7	0.8	37.2	42.9	44.4	44.4	44.6	44.6	44.6	35.5	35.5	42.9	42.9	32.4	22.9	22.9	14.6	37.2	39.7	39.7	39.7	42.4	48.8	36.9	36.2	36.2
143	77.9	110.9	93.9	89.7	89.4	98.9	75.0	77.3	77.3	93.0	89.9	70.6	91.0	91.0	86.2	86.2	86.2	86.1	86.1	94.1	94.1	58.5	63.3	63.3	57.4	89.9	95.8	95.8	95.8	92.4	86.3	57.7	49.3	49.3
144	77.4	65.4	73.5	79.5	62.4	53.1	57.0	41.7	41.7	39.7	75.5	84.7	69.3	69.3	80.8	80.8	80.8	76.9	76.9	76.0	76.0	41.9	69.8	69.8	60.5	75.5	81.0	81.0	81.0	80.0	76.3	74.3	55.8	55.8
145	44.8	37.9	37.9	38.9	40.2	43.0	33.7	19.4	19.4	0.1	36.7	38.8	41.5	41.5	40.7	40.7	40.7	33.1	33.1	36.8	36.8	23.8	3.5	3.5	0.9	36.7	38.4	38.4	38.4	41.1	43.5	31.3	29.0	29.0
146	82.9	65.0	68.7	69.4	75.2	96.3	92.1	89.2	89.2	6.1	65.3	71.8	68.9	68.9	72.3	72.3	72.3	64.8	64.8	72.8	72.8	57.5	70.1	70.1	55.5	65.3	74.9	74.9	74.9	76.5	62.6	61.0	99.6	99.6
147	72.9	66.1	65.0	69.9	69.8	76.0	83.4	86.2	86.2	27.0	64.9	78.3	76.1	76.1	75.1	75.1	75.1	66.4	66.4	72.6	72.6	55.2	72.3	72.3	60.6	64.9	68.4	68.4	68.4	67.9	71.5	57.7	66.1	66.1
148	40.0	34.1	35.8	36.9	38.2	40.9	40.7	30.2	30.2	0.1	33.8	37.8	38.8	38.8	38.4	38.4	38.4	31.3	31.3	37.2	37.2	23.8	11.3	11.3	3.9	33.8	36.4	36.4	36.4	38.7	44.0	34.5	30.8	30.8
149	81.7	97.4	98.3	83.1	68.0	85.4	64.8	76.9	76.9	40.8	78.0	67.0	96.4	96.4	94.2	94.2	94.2	79.8	79.8	93.1	93.1	60.7	59.2	59.2	53.1	78.0	93.2	93.2	93.2	92.4	87.0	62.0	52.7	52.7
150	36.1	27.9	28.0	28.4	29.4	28.0	21.9	6.1	6.1	0.3	28.4	28.3	31.7	31.7	31.1	31.1	31.1	22.8	22.8	26.9	26.9	19.2	0.4	0.4	0.2	28.4	27.8	27.8	27.8	33.6	33.0	25.0	17.5	17.5
151	72.5	74.5	82.8	73.1	58.0	80.6	58.7	43.3	43.3	29.8	77.0	50.9	78.9	78.9	90.5	90.5	90.5	77.1	77.1	66.9	66.9	45.6	83.7	83.7	76.6	77.0	75.9	75.9	75.9	94.4	89.1	87.4	65.7	65.7
152	37.2	31.6	32.1	33.0	34.6	39.4	37.8	29.8	29.8	0.2	31.9	36.8	33.5	33.5	34.2	34.2	34.2	29.0	29.0	31.8	31.8	23.0	12.8	12.8	4.2	31.9	35.1	35.1	35.1	37.1	38.4	27.1	24.4	24.4
153	86.2	77.8	82.5	79.4	66.4	91.1	102.2	80.4	80.4	89.4	77.1	71.1	91.1	91.1	94.4	94.4	94.4	73.3	73.3	82.2	82.2	51.1	119.3	119.3	116.7	77.1	86.1	86.1	86.1	93.1	97.2	106.9	143.7	143.7
154	83.5	70.2	82.3	84.6	70.2	82.7	53.5	58.0	58.0	62.3	93.6	99.0	79.1	79.1	91.3	91.3	91.3	84.2	84.2	84.6	84.6	49.8	68.5	68.5	61.5	93.6	82.9	82.9	82.9	81.9	81.5	78.6	67.8	67.8
155	70.6	59.0	56.3	64.6	71.5	76.2	81.1	81.7	81.7	7.2	57.3	68.8	70.1	70.1	66.3	66.3	66.3	59.6	59.6	67.0	67.0	53.4	55.3	55.3	33.5	57.3	67.8	67.8	67.8	66.9	69.7	62.1	67.7	67.7
156	84.8	92.0	86.7	87.3	70.7	89.4	72.6	54.5	54.5	39.6	83.2	80.3	85.2	85.2	84.6	84.6	84.6	76.2	76.2	86.3	86.3	65.5	69.5	69.5	56.8	83.2	88.5	88.5	88.5	93.6	79.2	76.5	67.2	67.2
157	79.9	79.5	78.5	78.4	77.6	94.2	78.4	66.6	66.6	90.5	80.6	77.6	93.3	93.3	93.2	93.2	93.2	75.2	75.2	86.6	86.6	71.8	100.0	100.0	91.4	80.6	86.4	86.4	86.4	91.3	175.0	132.8	106.3	106.3
158	79.2	82.2	78.1	78.5	68.0	72.5	81.1	82.9	82.9	75.3	81.2	68.4	76.0	76.0	73.0	73.0	73.0	74.4	74.4	74.0	74.0	61.3	72.7	72.7	65.2	81.2	79.5	79.5	79.5	81.7	93.4	73.5	58.9	58.9
159	76.9	75.9	76.1	75.4	75.3	80.7	92.5	78.6	78.6	102.8	74.8	74.5	82.1	82.1	80.5	80.5	80.5	69.2	69.2	82.1	82.1	60.2	94.7	94.7	90.9	74.8	86.2	86.2	86.2	83.0	107.7	99.4	89.4	89.4
160	79.0	89.9	89.7	80.4	72.1	77.8	79.8	79.7	79.7	117.7	92.1	70.3	88.2	88.2	91.0	91.0	91.0	79.5	79.5	86.4	86.4	80.0	71.2	71.2	69.7	92.1	80.2	80.2	80.2	85.3	91.3	80.3	73.5	73.5
161	102.5	270.7	202.8	129.3	85.1	198.0	279.8	232.6	232.6	256.4	169.3	56.1	195.7	195.7	123.3	123.3	123.3	87.9	87.9	88.0	88.0	17.8	43.0	43.0	49.6	169.3	89.7	89.7	89.7	138.8	507.4	174.3	236.5	236.5
162	90.4	81.8	82.5	92.1	85.1	86.4	78.6	64.2	64.2	76.6	94.5	84.7	98.6	98.6	97.4	97.4	97.4	89.2	89.2	93.8	93.8	67.7	99.4	99.4	96.2	94.5	95.2	95.2	95.2	102.5	141.1	141.3	117.2	117.2
163	99.3	103.3	102.7	99.1	102.6	126.1	89.9	110.9	110.9	160.8	104.0	90.7	83.9	83.9	87.5	87.5	87.5	95.8	95.8	87.3	87.3	81.2	99.7	99.7	94.7	104.0	97.7	97.7	97.7	100.4	111.7	108.3	115.7	115.7
164	75.0	86.1	90.5	78.8	76.2	75.5	74.9	88.0	88.0	64.3	87.5	73.3	85.0	85.0	85.1	85.1	85.1	75.7	75.7	84.6	84.6	75.4	74.3	74.3	66.0	87.5	81.6	81.6	81.6	79.8	81.8	82.7	78.3	78.3
165	75.2	79.4	75.6	73.5	76.0	84.0	72.6	74.3	74.3	44.2	78.9	67.4	84.0	84.0	80.3	80.3	80.3	68.6	68.6	77.1	77.1	54.6	85.8	85.8	79.8	78.9	85.0	85.0	85.0	88.2	98.2	85.7	70.5	70.5
166	81.5	89.0	75.2	86.7	70.2	74.8	60.2	31.3	31.3	28.8	77.6	68.5	90.7	90.7	79.1	79.1	79.1	69.2	69.2	76.3	76.3	20.8	51.6	51.6	54.6	77.6	78.3	78.3	78.3	84.7	237.4	213.6	170.0	170.0
167	72.5	76.9	71.1	72.1	58.3	71.5	70.0	63.1	63.1	46.3	74.2	60.0	83.3	83.3	82.9	82.9	82.9	67.5	67.5	81.8	81.8	61.5	74.4	74.4	64.9	74.2	75.4	75.4	75.4	76.4	94.7	82.4	84.8	84.8
168	86.1	83.6	83.9	88.4	73.7	90.1	81.4	70.5	70.5	137.1	86.5	65.8	92.5	92.5	90.1	90.1	90.1	81.9	81.9	85.6	85.6	64.4	104.0	104.0	94.6	86.5	90.4	90.4	90.4	96.4	103.5	91.6	80.7	80.7
169	84.9	84.3	86.2	84.9	66.9	84.6	80.3	75.9	75.9	87.5	97.4	73.9	100.3	100.3	96.7	96.7	96.7	77.2	77.2	89.0	89.0	72.9	102.3	102.3	92.3	97.4	88.1	88.1	88.1	94.3	123.6	110.3	106.1	106.1
170	87.4	89.1	78.6	89.1	62.3	92.4	105.3	93.9	93.9	94.8	79.3	84.4	94.0	94.0	83.2	83.2	83.2	83.1	83.1	94.4	94.4	63.0	108.9	108.9	93.1	79.3	90.2	90.2	90.2	87.2	251.2	179.1	115.2	115.2
171	81.3	77.9	80.8	78.9	66.4	75.9	61.6	46.2	46.2	12.7	85.7	82.5	84.8	84.8	87.1	87.1	87.1	74.4	74.4	81.7	81.7	64.2	61.5	61.5	46.3	85.7	82.1	82.1	82.1	85.9	74.9	74.3	72.1	72.1
172	78.9	87.1	79.6	80.9	68.7	75.6	70.4	54.7	54.7	54.0	89.1	64.0	85.8	85.8	88.3	88.3	88.3	72.2	72.2	82.4	82.4	60.8	74.3	74.3	66.8	89.1	77.4	77.4	77.4	84.2	83.1	88.5	98.6	98.6
173	83.1	89.6	90.0	86.8	86.2	83.9	86.5	74.6	74.6	49.3	77.5	65.5	89.2	89.2	85.1	85.1	85.1	79.0	79.0	81.4	81.4	60.2	76.2	76.2	69.9	77.5	90.1	90.1	90.1	92.9	75.6	103.6	67.9	67.9
174	73.2	73.1	71.7	72.1	71.5	64.7	59.8	54.2	54.2	111.4	88.4	81.3	73.8	73.8	74.2	74.2	74.2	69.3	69.3	70.0	70.0	49.0	65.6	65.6	89.4	88.4	75.7	75.7	75.7	74.6	162.0	178.4	147.0	147.0
175	83.2	107.3	92.0	76.8	81.3	109.4	105.9	82.4	82.4	149.9	85.6	65.7	101.7	101.7	81.9	81.9	81.9	68.7	68.7	85.5	85.5	36.4	73.1	73.1	92.2	85.6	88.6	88.6	88.6	82.4	289.8	274.7	223.6	223.6
176	88.4	87.1	89.4	90.0	80.7	92.9	79.7	73.5	73.5	59.2	104.1	90.3	86.5	86.5	88.2	88.2	88.2	84.7	84.7	91.3	91.3	74.6	76.3	76.3	66.6	104.1	87.4	87.4	87.4	89.6	83.2	80.1	79.2	79.2
177	82.7	84.6	84.2	79.4	72.3	83.8	66.0	72.6	72.6	136.4	89.9	87.2	93.6	93.6	96.9	96.9	96.9	76.3	76.3	89.0	89.0	63.3	74.3	74.3	110.8	89.9	81.8	81.8	81.8	83.1	172.2	185.1	128.8	128.8
178	86.0	112.9	109.0	86.7	89.1	124.0	110.2	91.9	91.9	86.0	87.2	74.8	88.3	88.3	90.0	90.0	90.0	88.0	88.0	84.2	84.2	64.7	83.9	83.9	71.0	87.2	92.4	92.4	92.4	93.1	79.5	83.1	78.7	78.7
179	89.0	98.2	97.6	84.3	72.8	96.2	82.0	88.9	88.9	97.3	85.0	67.4	84.3	84.3	86.6	86.6	86.6	87.2	87.2	80.7	80.7	68.8	88.2	88.2	101.2	85.0	92.5	92.5	92.5	97.1	90.9	125.1	78.3	78.3
180	88.0	95.2	96.0	88.5	83.9	101.3	98.6	113.3	113.3	47.6	95.8	70.5	87.9	87.9	92.9	92.9	92.9	84.5	84.5	88.7	88.7	80.3	84.3	84.3	72.5	95.8	89.7	89.7	89.7	95.0	86.2	104.0	103.6	103.6
181	80.7	95.5	101.0	82.7	99.0	100.3	82.5	81.2	81.2	79.2	86.3	88.7	81.9	81.9	81.2	81.2	81.2	82.6	82.6	88.4	88.4	68.2	77.0	77.0	71.3	86.3	83.9	83.9	83.9	82.0	80.2	86.3	88.9	88.9
182	87.0	80.7	85.7	87.1	73.2	79.4	83.8	87.2	87.2	78.1	90.9	78.5	91.5	91.5	91.0	91.0	91.0	85.3	85.3	97.5	97.5	82.2	87.5	87.5	82.5	90.9	84.4	84.4	84.4	90.0	83.2	88.4	95.6	95.6
183	80.3	80.9	78.4	82.8	75.1	67.5	85.3	63.8	63.8	32.0	84.4	66.3	90.3	90.3	90.8	90.8	90.8	78.0	78.0	84.0	84.0	74.0	73.7	73.7	60.0	84.4	83.6	83.6	83.6	86.5	82.4	89.8	84.2	84.2
184	82.7	95.0	96.0	84.5	81.8	89.4	93.2	65.7	65.7	74.8	93.0	86.8	88.4	88.4	88.7	88.7	88.7	82.8	82.8	87.5	87.5	75.4	81.8	81.8	65.6	93.0	88.6	88.6	88.6	90.2	85.0	83.5	69.9	69.9
185	77.1	73.4	75.6	78.9	82.2	71.4	56.1	53.2	53.2	29.2	90.2	79.0	82.1	82.1	83.7	83.7	83.7	70.6	70.6	76.8	76.8	55.3	64.9	64.9	46.1	90.2	82.9	82.9	82.9	80.6	75.7	83.5	91.6	91.6
186	74.1	77.4	76.2	72.3	70.6	71.0	73.5	52.4	52.4	8.8	71.5	67.5	73.8	73.8	70.3	70.3	70.3	66.7	66.7	66.8	66.8	45.7	53.8	53.8	31.6	71.5	75.3	75.3	75.3	77.6	76.2	75.5	67.3	67.3
187	80.2	82.2	77.7	79.4	65.3	76.7	77.2	69.0	69.0	135.6	81.9	62.0	83.8	83.8	85.7	85.7	85.7	74.7	74.7	84.1	84.1	80.7	77.1	77.1	74.3	81.9	82.1	82.1	82.1	85.6	121.9	81.8	86.4	86.4
188	89.6	89.1	91.2	84.9	81.8	84.7	65.5	72.1	72.1	74.1	80.8	78.2	97.5	97.5	102.8	102.8	102.8	82.9	82.9	97.7	97.7	60.7	71.0	71.0	94.1	80.8	91.9	91.9	91.9	90.3	167.2	129.3	113.8	113.8
189	81.1	87.1	83.2	80.9	81.7	83.8	85.3	92.7	92.7	107.4	87.7	86.7	81.1	81.1	77.6	77.6	77.6	75.7	75.7	79.9	79.9	68.1	86.3	86.3	70.7	87.7	88.5	88.5	88.5	90.0	72.3	52.6	62.8	62.8
190	82.8	102.9	86.5	87.9	75.3	102.7	69.9	44.0	44.0	49.1	87.6	77.5	111.0	111.0	102.2	102.2	102.2	80.9	80.9	95.5	95.5	63.1	87.8	87.8	78.3	87.6	95.0	95.0	95.0	95.2	94.0	69.9	61.6	61.6
191	74.3	75.2	76.8	81.1	56.4	50.8	40.2	29.5	29.5	41.8	89.6	53.9	71.4	71.4	83.4	83.4	83.4	78.1	78.1	77.8	77.8	44.3	67.7	67.7	49.3	89.6	79.6	79.6	79.6	81.9	78.1	77.8	59.1	59.1
192	93.3	101.6	104.6	99.3	87.8	101.9	78.8	69.8	69.8	57.8	100.6	75.0	91.2	91.2	111.0	111.0	111.0	95.7	95.7	99.3	99.3	67.2	80.8	80.8	65.7	100.6	107.1	107.1	107.1	109.8	102.0	106.4	71.4	71.4
193	80.9	82.9	87.0	84.7	81.1	79.5	61.3	29.4	29.4	37.7	78.2	73.7	87.1	87.1	94.3	94.3	94.3	81.5	81.5	79.9	79.9	54.7	69.2	69.2	65.4	78.2	69.6	69.6	69.6	74.6	101.1	87.5	89.8	89.8
194	86.8	79.8	83.9	92.4	78.4	69.3	63.5	82.0	82.0	74.5	87.7	74.3	79.0	79.0	100.5	100.5	100.5	83.0	83.0	89.3	89.3	69.5	81.4	81.4	63.0	87.7	93.0	93.0	93.0	94.9	98.6	93.8	81.1	81.1
195	83.8	87.5	94.1	87.4	86.3	91.1	54.7	31.6	31.6	73.1	85.3	68.2	84.7	84.7	97.2	97.2	97.2	85.9	85.9	83.2	83.2	44.8	68.1	68.1	56.6	85.3	91.9	91.9	91.9	95.8	92.4	84.0	65.2	65.2
196	78.9	95.8	99.2	85.5	79.9	97.9	83.8	73.1	73.1	56.5	97.1	80.4	66.7	66.7	80.9	80.9	80.9	84.0	84.0	75.1	75.1	50.6	81.5	81.5	67.4	97.1	97.6	97.6	97.6	97.5	90.5	80.0	58.5	58.5
197	81.5	71.7	85.5	86.1	72.6	75.2	58.7	57.1	57.1	34.1	91.9	77.0	86.7	86.7	104.3	104.3	104.3	87.3	87.3	96.9	96.9	67.2	69.4	69.4	59.7	91.9	85.5	85.5	85.5	88.8	103.9	93.0	71.6	71.6
198	84.0	81.4	88.4	90.4	91.2	101.7	84.1	67.3	67.3	33.6	89.8	80.9	78.5	78.5	95.9	95.9	95.9	88.9	88.9	91.3	91.3	66.2	86.6	86.6	77.2	89.8	94.2	94.2	94.2	98.7	142.8	139.6	113.2	113.2
199	81.2	97.0	98.9	88.3	78.0	111.4	76.0	69.7	69.7	71.6	82.8	74.3	89.7	89.7	110.6	110.6	110.6	84.9	84.9	93.8	93.8	60.6	85.6	85.6	70.3	82.8	89.6	89.6	89.6	101.4	65.2	72.4	66.2	66.2
200	73.2	82.3	86.0	79.6	61.8	60.8	42.2	18.7	18.7	60.2	96.1	63.6	77.6	77.6	95.0	95.0	95.0	76.8	76.8	84.0	84.0	38.9	60.1	60.1	45.1	96.1	76.2	76.2	76.2	79.5	91.7	84.6	67.1	67.1
201	69.3	77.4	93.7	75.8	66.9	59.5	33.7	24.9	24.9	56.9	85.1	63.1	84.3	84.3	92.5	92.5	92.5	71.4	71.4	81.3	81.3	43.6	55.7	55.7	45.0	85.1	71.7	71.7	71.7	76.0	96.4	95.0	67.2	67.2
202	90.1	84.1	92.9	93.3	56.8	80.1	66.4	49.1	49.1	68.8	101.4	67.5	105.2	105.2	115.1	115.1	115.1	96.0	96.0	96.2	96.2	69.3	86.3	86.3	68.8	101.4	92.9	92.9	92.9	103.6	114.5	103.1	86.3	86.3
203	83.4	78.7	85.1	84.3	86.9	67.4	58.0	46.0	46.0	64.8	80.6	86.8	91.0	91.0	94.0	94.0	94.0	82.1	82.1	81.6	81.6	58.7	79.9	79.9	77.9	80.6	81.5	81.5	81.5	85.6	109.8	84.9	81.3	81.3
204	86.0	83.1	74.0	89.9	77.1	88.8	79.3	54.4	54.4	89.8	74.3	88.9	77.2	77.2	79.1	79.1	79.1	87.7	87.7	76.6	76.6	62.6	94.5	94.5	83.0	74.3	86.7	86.7	86.7	91.7	98.3	69.8	79.0	79.0
205	84.1	84.6	80.0	87.2	97.6	75.5	53.9	40.7	40.7	70.4	73.6	97.4	85.4	85.4	88.7	88.7	88.7	83.1	83.1	87.9	87.9	50.7	83.8	83.8	76.1	73.6	82.4	82.4	82.4	86.1	118.6	101.4	94.5	94.5
206	85.2	77.5	77.3	85.8	76.8	78.9	61.2	42.4	42.4	22.7	78.8	89.7	75.7	75.7	78.5	78.5	78.5	81.2	81.2	76.4	76.4	53.3	78.4	78.4	71.7	78.8	79.9	79.9	79.9	84.9	96.0	75.6	69.3	69.3
207	77.2	74.6	79.0	78.3	82.2	91.4	69.8	51.4	51.4	79.9	84.2	81.2	76.8	76.8	78.4	78.4	78.4	76.8	76.8	73.5	73.5	49.8	73.4	73.4	66.3	84.2	74.1	74.1	74.1	78.9	108.2	86.5	76.8	76.8
208	75.0	86.2	84.9	82.1	70.1	69.4	61.2	43.6	43.6	53.4	93.8	80.0	72.7	72.7	89.4	89.4	89.4	81.8	81.8	75.6	75.6	51.0	80.8	80.8	70.9	93.8	84.2	84.2	84.2	91.2	79.1	74.4	66.5	66.5
209	76.9	69.3	85.5	79.1	67.5	66.0	53.2	35.9	35.9	20.2	86.6	82.6	82.2	82.2	90.5	90.5	90.5	78.9	78.9	87.7	87.7	48.1	65.8	65.8	52.1	86.6	88.5	88.5	88.5	89.8	86.5	78.9	73.8	73.8
210	84.8	76.2	85.6	89.5	85.9	87.3	80.0	62.1	62.1	94.4	87.5	88.9	79.2	79.2	87.0	87.0	87.0	86.7	86.7	85.4	85.4	58.7	79.8	79.8	64.8	87.5	78.2	78.2	78.2	83.7	139.5	97.0	86.5	86.5
211	77.7	88.3	100.1	79.4	80.7	72.0	62.8	39.8	39.8	65.5	88.6	61.2	84.3	84.3	100.8	100.8	100.8	79.2	79.2	93.7	93.7	65.2	84.4	84.4	68.8	88.6	83.9	83.9	83.9	88.0	94.9	97.4	74.7	74.7

Section S6. PFAS Method Detection Limits.

Table S6. Human Liver Specimen Method Detection Limits (MDL). The method detection limit (MDL) is defined as the blank average plus three times the standard deviation. In cases where the method detection limit was below the instrumental detection limit, this value was replaced with the instrumental detection limit. Only samples that were above MDL values are reported in the manuscript.

Analyte	Instrument Detection Limit [ng/sample]	Method Detection Limit [ng/g]
PFPrA	0.003	0.0321
PFBA	0.007	0.0895
PFPeA	0.049	0.1154
PFHxA	0.009	0.0887
PFHpA	0.044	0.2374
PFOA	0.001	0.0824
PFNA	0.04	0.0942
PFDA	0.05	0.1178
PFUdA	0.03	0.0909
PFDoA	-	-
PFTrDA	0.019	0.0448
PFTeDA	0.001	0.0024
3:3 FTCA	-	-
5:3 FTCA	0.034	0.0801
9:3 FTCA	0.001	-
FOEA	4.539	10.692
PFPrS	0.038	0.0895
PFBS	0.06	0.1413
PFPeS	0.052	0.1225
L-PFHxS	0.001	0.0462
Br-PFHxS	0.004	0.0094
PFHpS	0.053	0.1248
L-PFOS	0.001	0.0298
Br-PFOS	0.001	0.0334
PFNS	0.108	0.2544
PFDS	0.011	0.0259
MeFBSA	0.302	0.7114
N-EtFBSA-M	0.487	1.1471
FBSA	0.045	0.106
FPeSA	0.028	0.066
FHxSA	0.031	0.073
FHpSA	0.003	0.0071
FOSA	0.004	0.0454
L-N-MeFOSAA	0.056	0.1319
Br-N-MeFOSAA	0.113	0.2662
L-N-EtFOSAA	0.125	0.2944

Br-N-EtFOSAA	1.431	3.3707
MeFOSA	0.001	0.0024
EtFOSA	0.001	0.0024
HFPO-DA	0.137	0.3227
DONA	0.003	0.0071
9ClPF3ONS	0.008	0.0188
11ClPF3OUdS	0.001	0.0024
3,6-OPFHpA	0.015	0.0713
PF4OPeA	0.024	0.0565
PF5OHxA	0.026	0.0612
PFECHS	0.037	0.0872
PFEESA	0.049	0.1154
N-AP-FHxSA	0.002	0.0047
N-TAmP-FHxSA	-	-
4:2 FTS	0.001	0.0024
6:2 FTS	0.003	0.1067
8:2 FTS	0.005	0.0118
10:2 FTS	0.19	0.4475

Section S7. Extractable Organofluorine Analysis QC/QA.

Table S7. Extractable Organofluorine Analysis QC/QA. Positive controls were spiked with a PFAS mixture (9,948 ng F/mL PFAC-24; matrix-matched bovine liver, n = 4; procedural spike, n = 2), and negative controls were spiked with sodium fluoride (10 mg F/L; inorganic fluorine controls, n = 4). Organic fluorine recoveries indicate that the extraction controls analyzed passed QC/QA.

Samples	Sample Type	Inorganic Fluorine (IF) Spike	Organic Fluorine (OF) Spike	OF Recovery (%)
Procedural Spike 1	DI Water		25 uL x 9948 ng F/mL PFAS	70
Procedural Spike 2	DI Water		25 uL x 9948 ng F/mL PFAS	53
Matrix Liver OF Low Spike 1	Bovine Liver		25 uL x 9948 ng F/mL PFAS	74
Matrix Liver OF Low Spike 2	Bovine Liver		25 uL x 9948 ng F/mL PFAS	69
Matrix Liver OF High Spike 1	Bovine Liver		100 uL x 9948 ng F/mL PFAS	87
Matrix Liver OF High Spike 2	Bovine Liver		100 uL x 9948 ng F/mL PFAS	80
Matrix Liver IF Spike 1	Bovine Liver	100 uL x 10 mg F/L NaF		-
Matrix Liver IF Spike 2	Bovine Liver	100 uL x 10 mg F/L NaF		-
Matrix Liver IF Spike 3	Bovine Liver	100 uL x 10 mg F/L NaF		-
Matrix Liver IF Spike 4	Bovine Liver	100 uL x 10 mg F/L NaF		-

Section S8. EOF Method Detection Limits.

Table S8. Human Liver Specimen EOF Method Detection Limits (MDL). The method detection limit (MDL) is defined as three times the standard deviation of the matrix extraction blanks, adjusted by the dilution factor. Only samples that were above MDL values are reported in the manuscript.

EOF Method Detection Limits (MDL) [ng F/g]	
Batch 1	104.75
Batch 2	91.54

Section S9. EOF and Targeted PFAS Analysis Donor Specimen Demographics.

Table S9. EOF and targeted PFAS analysis donor specimen demographics (N=17). EOF and targeted PFAS concentrations were matched by donor demographics.

	**EOF Specimen (n=8)**	**Targeted PFAS Specimen (n=11)**
**Age mean ± SD (Range)**	42.50 ± 13.97 (20-62)	48.82 ± 23.54 (20-62
**Gender, n (%)**		
Male	6 (75%)	8 (73%)
Female	2 (25%)	3 (27%)
**Ethnicity, n (%)**		
Other/Unknown	8 (100%)	11 (100%)
**Collection Year Range, n (%)**	2000 (8, 100%)	2000 (11, 100%)
**BMI[a] (kg/m²), mean ± SD (Range, n)**	Unknown	Unknown
**Health Status, n (%)**		
Normal	8 (100%)	8 (100%)
**Tissue Bank Specimen Location, donor n (%)**		
LTCDS[b]	MN, 8 (100%)	MN, 11 (100%)

[a] BMI = Body mass index
[b] LTCDS = Liver Tissue Cell Distribution System, University of Minnesota

Section S10. Weighted Linear Regression Plots.

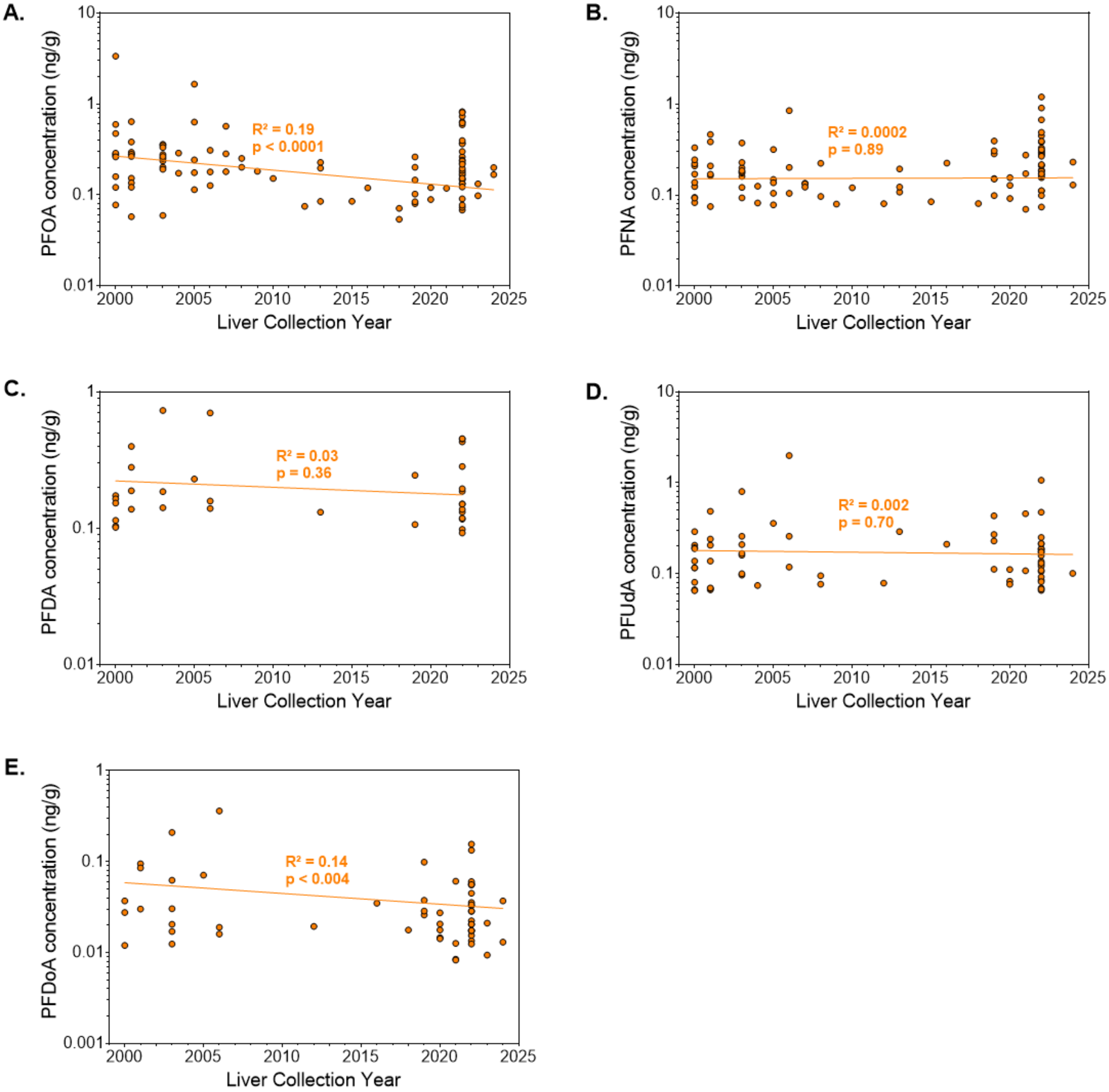

Figure S2. Temporal trends in the concentration of perfluoroalkyl carboxylic acids (PFCAs) in human liver samples collected from 2000 to 2024. Each point represents an individual liver sample with an exact sampling year recorded. The y-axis represents a PFCA compound detected per sample (ng/g frozen weight) on a logarithmic scale. A weighted linear regression was performed on log-transformed perfluoroalkyl carboxylic acids - perfluorooctanoic acid [PFOA (*n=99*), S1.A], perfluorononanoic acid [PFNA (*n=91*), S1.B], perfluorodecanoic acid [PFDA (*n=34*), S1.C], perfluoroundecanoic acid [PFUdA (*n=67*), S1.D], and perfluorododecanoic acid [PFDoA (*n=57*), S1.E]. The detection frequency of these PFCAs ranged from 34 to 99 livers. The slope of the regression (R^2) indicates PFAS concentration trends over time, and the p-value denotes the significance of the data results.

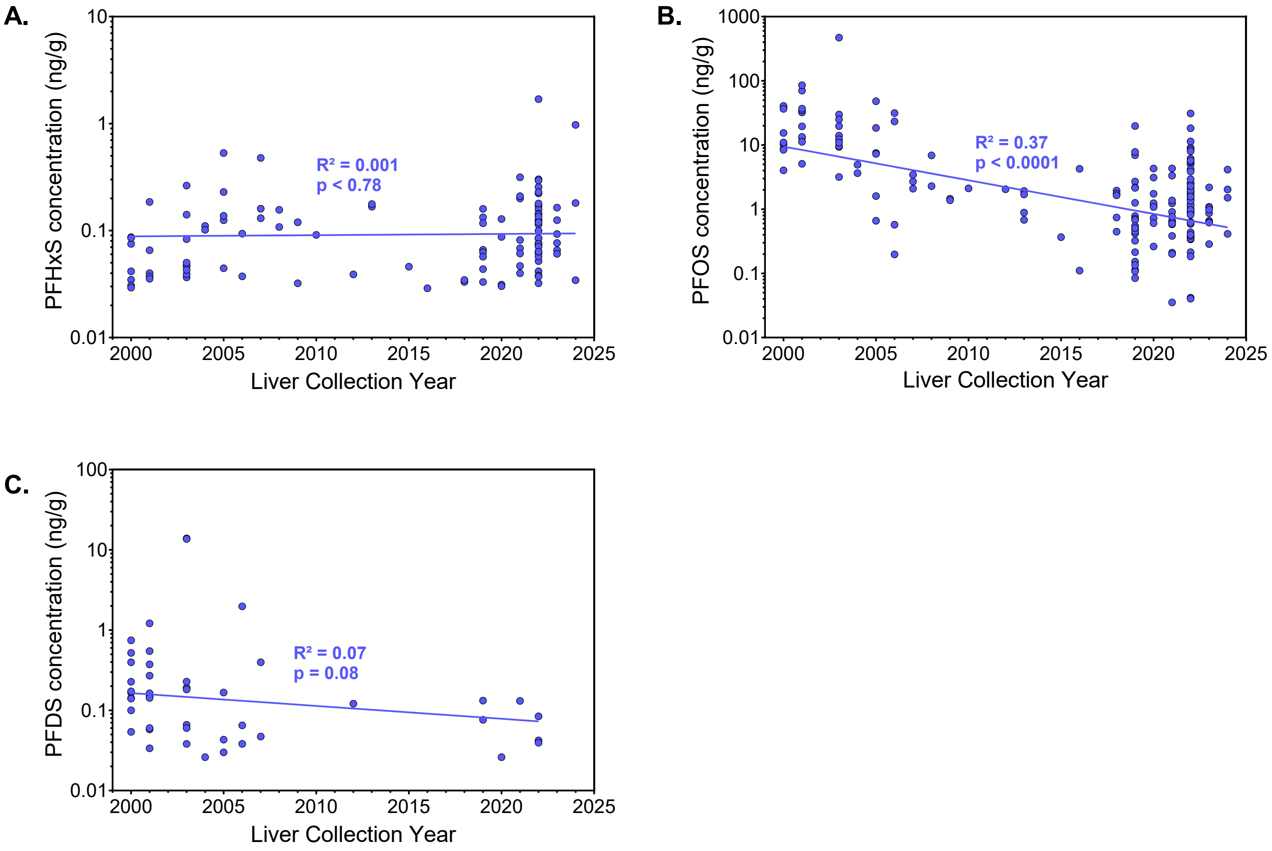

Figure S3. Temporal trends in the concentration of perfluoroalkyl sulfonic acids (PFSAs) in human liver samples collected from 2000 to 2024. Each point represents an individual liver sample with an exact sampling year recorded. The y-axis represents a PFSA compound detected per sample (ng/g frozen weight) on a logarithmic scale. A weighted linear regression was performed on log-transformed perfluoroalkyl sulfonic acids - perfluorohexanesulfonic acid [∑PFHxS linear and branched isomers (*n=106*), S2.A], perfluorooctanesulfonic acid [∑PFOS linear and branched isomers (*n=176*), S2.B], and perfluorodecanesulfonic acid [PFDS (*n=46*), S2.C]. The detection frequency of these PFSAs ranged from 46 to 176 livers. The slope of the regression (R^2) indicates PFAS concentration trends over time, and the p-value denotes the significance of the data results.

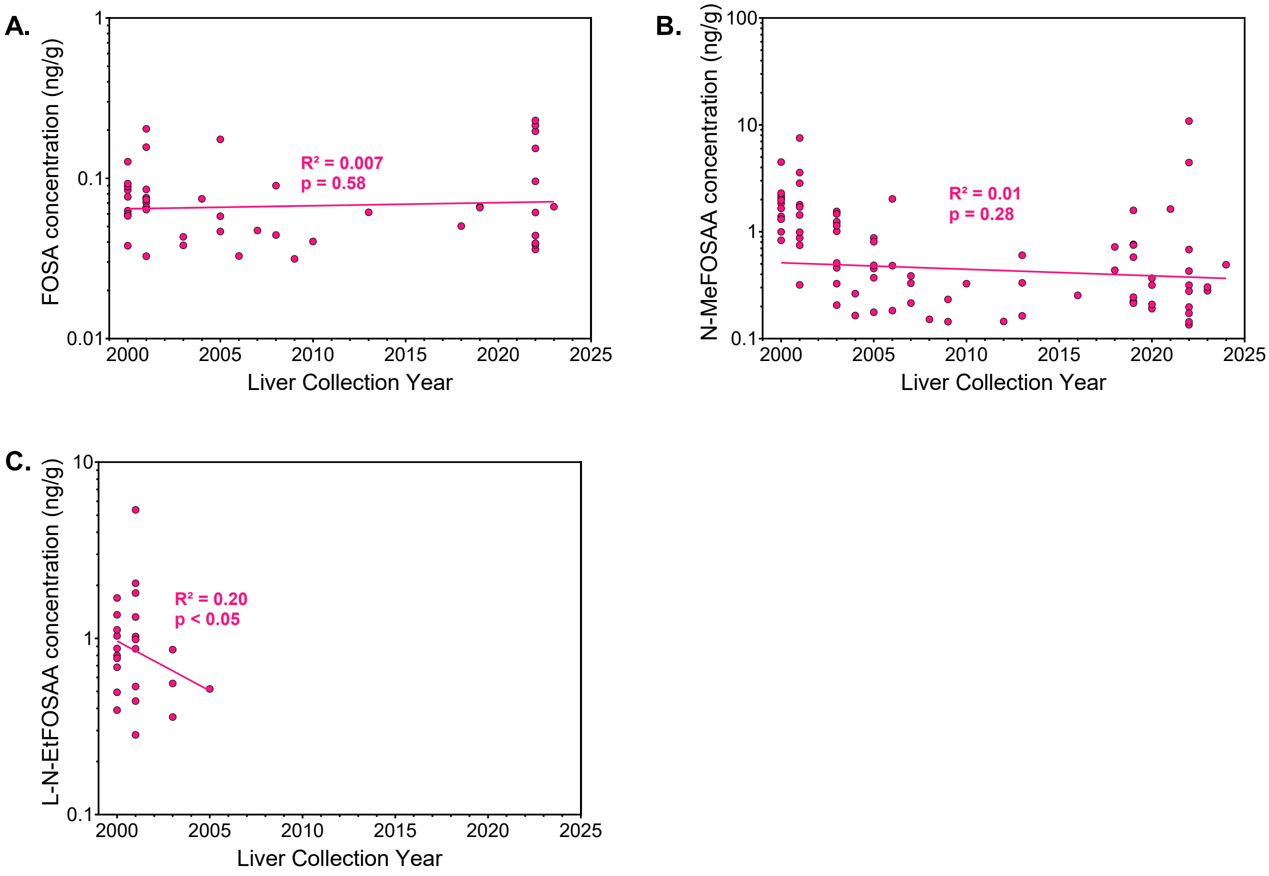

Figure S4. Temporal trends in the concentration of perfluorooctanesulfonamides and derivatives (FOSAA) in human liver samples collected from 2000 to 2024. Each point represents an individual liver sample with an exact sampling year recorded. The y-axis represents a FOSAA compound detected per sample (ng/g frozen weight) on a logarithmic scale. A weighted linear regression was performed on log-transformed perfluorooctanesulfonamides and derivatives – Perfluorooctanesulfonamide [FOSA (*n=45*), S3.A], N-methyl perfluorooctanesulfonamidoacetic acid [∑N-MeFOSAA linear and branched isomers (*n=81*), S3.B], and linear N-ethyl perfluorooctanesulfonamidoacetic acid [L-N-EtFOSAA (*n=24*), S2.C]. The detection frequency of these FOSAAs ranged from 24 to 81 livers. The slope of the regression (R^2) indicates PFAS concentration trends over time, and the p-value denotes the significance of the data results.

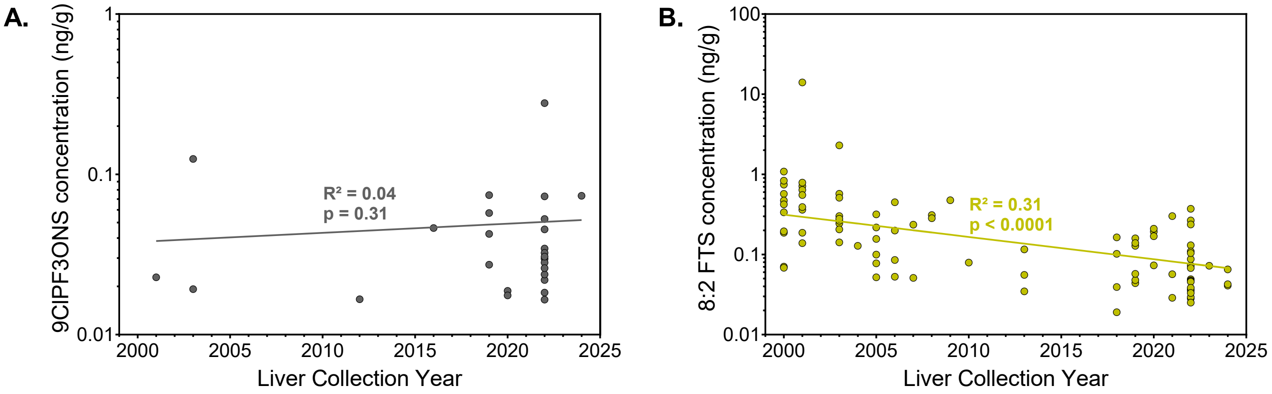

Figure S5. Temporal trends in the concentration of chlorinated ethers and fluorotelomer sulfonates in human liver samples collected from 2000 to 2024. Each point represents an individual liver sample with an exact sampling year recorded. The y-axis represents either a chlorinated ether or a fluorotelomer sulfonate compound detected per sample (ng/g frozen weight) on a logarithmic scale. A weighted linear regression was performed on log-transformed chlorinated ethers and fluorotelomer sulfonates– 9-chlorohexadecafluoro-3-oxanonane-1-sulfonic acid (9Cl-PF3ONS (*n=27*), S3.A], and 8:2 Fluorotelomer sulfonic acid [8:2 FTS (*n=92*), S4.B]. The slope of the regression (R^2) indicates PFAS concentration trends over time, and the p-value denotes the significance of the data results.

Section S11. Multivariate Linear Regression Analysis.

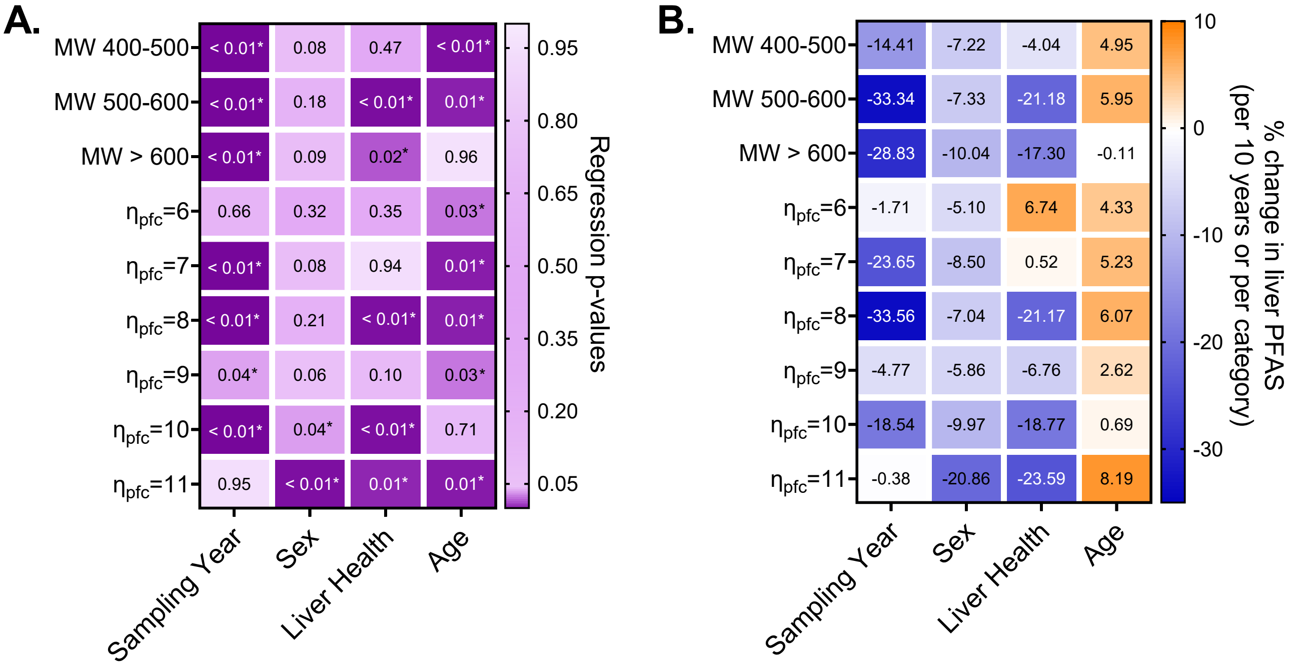

Figure S6. Results of multivariate linear regression analysis for PFAS detected in ≥30 of the 211 human liver samples collected from 2000 to 2024 (n = 205 with complete metadata) categorized based on molecular weight (g/mol) range and total number of perfluorinated carbons (η_{pfc}). Panel A shows regression p-values (purple scale), with dark purple cells indicating statistical significance ($p < 0.05$, asterisk) and lighter purple tones representing higher p-values. Panel B shows regression β estimates transformed into percent change in liver PFAS concentrations per 10-year increase in sampling year or age, or per category shift in sex (male to female) or liver health status (healthy to NAFLD). Positive associations are shown in orange, and negative associations in blue. Models were based on log_{10}-transformed PFAS concentrations; non-detects were replaced with MDL/2 prior to transformation.
